# Enhancement of Nutrient, Trace Element, and Organic Selenium Contents of Ratooning Rice Grains and Straw Through Foliar Application of Selenite

**DOI:** 10.3390/foods13223637

**Published:** 2024-11-14

**Authors:** Wenjiang Wu, Deqiang Qi, Yalong Chen, Jiaqi Wang, Qinghua Wang, Yanjun Yang, Hongbin Niu, Quanzhi Zhao, Ting Peng

**Affiliations:** 1 Innovation Center of Henan Grain Crops, Henan Key Laboratory of Rice Biology, Henan Agricultural University, Zhengzhou 450046, China; wuwenjiang1985@126.com (W.W.); bayiqdq@163.com (D.Q.); 15236127252@163.com (Y.C.); jq50312@outlook.com (J.W.); 2 College of Agronomy, Henan Agricultural University, Zhengzhou 450046, China; newhobby@163.com; 3 Forestry and Fruit Research Institute, Beijing Academy of Agricultural Sciences, Beijing 100089, China; qinghuasummer@163.com; 4 Fruit Tree Research Institute, Shanxi Agricultural University, Taiyuan 030031, China; yangyanjun0634@163.com; 5 College of Agronomy, Guizhou University, Guiyang 550025, China

**Keywords:** RR, biofortification, selenite, functional component content, nutritional quality

## Abstract

Selenium (Se) is an essential trace element that has various beneficial effects for human healthy. However, the effects of different Se forms and concentrations on growth and development, photosynthetic characteristics and antioxidant capacity are still unclear with regard to the dual grain-and-feed dual-use of ratoon rice (RR). In this study, three concentrations of three different Se forms were applied to RR using the foliar spraying method, and the results showed that Se treatment can increase the Se content of rice grain and straw. All the Se treatments improved the photosynthetic indexes and activities of antioxidant enzymes. The Se and trace elements contents, and the percentages of organic Se and protein Se of brown rice were found to be similar in all three Se forms. A higher organic Se content was found in the grain by spraying sodium selenite and Se-Met, in which the resistant starch (RS) content was increased with the increase in amylose content in grains. The main Se species in the grain was SeMet and the SeMeCys was found only with SeMet treatments. The grain quality showed that all three Se forms increased the consistency of gelatinization. Our study indicated that exogenous Se could improve the nutritional quality of both grain and straw by improving photosynthetic traits and antioxidant enzyme activities, especially sodium selenite and Se-Met. These results underscore the potential of foliar biofortification to enhance the functional component contents of RR grains and provide an insight into the Se enrichment of ratoon rice.

## 1. Introduction

Selenium (Se) is an important mineral nutrient element in plants. The importance of the plant system has been established through various studies in recent times, and Se is concomitant with the antioxidant activities in plants, significantly strengthening their resistance to a varied range of abiotic stressors such as heavy metals or metalloids, salinity, cold, ultraviolet (UV) exposure, and drought [1]. Previous studies showed that low concentrations of Se promoted the growth of plants, but high concentrations of Se induced oxidative damage in plants [2,3]. The deficiency of Se in the human diet results in health disorders such as muscle syndrome, Keshan disease, liver disease, cognitive impairment and many cancers [4]. Certain regions of the world are Se-deficient while others are becoming Se-toxic due to natural and anthropogenic activities [5]. Both the problems relating to Se i.e., deficiency and toxicity are harmful to humans and animals, hence, all over the world it is regarded as a two edged sword [6]. The World Health Organization (WHO) has recommended 50–55 µg·day^−1^ Se in human diet all over the world [7]. In humans, Se deficiency occurs when the dietary intake of Se is (<40 µg·day^−1^) and chronic toxicity is observed above levels of (>400 µg·day^−1^) [4]. In livestock, the minimal requirement of Se is 0.05–0.10 mg·kg^−1^·day^−1^ forage, while a toxic Se concentration in animal feed is 2–5 mg·kg^−1^·dry^−1^ forage [8]. Keshan and Kashin Beck are severe Se deficiency diseases reported in China due to a low intake of Se in the diet at a level of 7–11 µg·day^−1^ [9]. Plants are the main source of dietary Se for human beings and animals; hence, knowledge about Se compounds in plants is crucial [9].

Rice is a primary Se dietary source for the population, with humans relying on it as a staple food. One study showed that 75 percent of rice samples contained Se levels that were insufficient to meet human health needs [10]. Producing Se-enriched rice through the application of Se can help improve human Se deficiency and has nutritional, health, and disease-preventative effects [11,12]. This is a highly anticipated functional food on the modern consumer market. In order to produce functional foods such as Se-enriched rice, there are currently two approaches being explored for the Se-enrichment of rice. One is to produce Se-enriched offspring through the genetic combination of multiple Se-enriched parents [13,14], but this breeding process is slow with a low efficiency, and can be listed as a long-term plan for the Se-enrichment industry. The other is the enhancement of the Se content in rice grains by applying exogenous inorganic Se to the leaves or the soil [12]. The success of soil application depends largely on the homogeneity of the physicochemical properties of the soil, including soil structure and soil pH [15]. But its side effects are obvious, including soil fixation, runoff with rainwater erosion, and environmental pollution. In fact, foliar spraying eliminates the transportation of Se from the roots to the ground and transports it directly from the leaves to the grains, which is more bioavailable than soil-applied Se. The Se enrichment effect by foliar spraying is eight times higher than that of the soil-applied method [16]. Hence, the foliar spraying method could promote the uptake and accumulation of Se in the straw and grains of rice, making it beneficial for the utilization in both functional foods and animal feed [13,14].

Rice is the main grain crop in China. It is not only the main source of dietary calories, but also a key source of Se intake [17]. However, in major rice-producing regions worldwide, the average Se concentration in rice is only 95 μg·kg^−1^ [10]. Therefore, increasing the Se concentration in rice through the application of Se fertilizer is highly important for improving human Se nutrition [11]. Plants can produce selenoproteins via inorganic Se compounds, and the use of inorganic Se for biological enhancement is more cost-effective because organic Se compounds are expensive [18]. Therefore, owing to the high market price of Se-enriched rice, the cultivation of Se-enriched rice has rapidly increased. The application of Se to increase the Se content in rice and increase human Se intake through the diet is recognized as the most direct, effective, economical, and safe method of Se supplementation worldwide [1,10,16,17].

Ratooning rice (RR) is a method that utilizes the regenerative characteristics of rice to promote the germination of dormant buds on rice piles after harvest through cultivation and management measures, and further grows and develops into a short-growth-period rice [19]. This model can increase production and income, save planting and labor, and reduce fertilizer and pesticide usage [20]. RR has become an important simplified planting model in the southern rice-growing areas of China, and the development of RR is highly important for increasing grain yield, improving farmers’ income, and promoting stable agricultural development [21], and playing an increasingly important role in ensuring global food security and stability [22,23]. As a two-harvest regenerated rice cultivation model, although it has the advantages of reducing labor costs, alleviating agricultural busyness, and reducing labor intensity with the main purpose of high-quality grain production, it has a disadvantage of the poor quality of rice in the current season, which leads to a low commodity value of the grain. The reason for this is due to the higher environmental temperature during the current season of grain filling, which results in a decrease in processing, appearance, and cooking taste quality [24,25,26]. Research has shown that high temperatures during the grain filling and fruiting stage of rice accelerate the grain filling rate, resulting in a less compact arrangement of starch granules, increased chalkiness, and a decrease in amylose content [27]. Owing to the impact of global climate change, the increasing frequency of extreme temperatures will further threaten the production of high-quality rice. Further research suggests that the quality of rice produced in the subsequent season is not only better than that of the current-season rice, but also better than that of late-season rice of the same genotype that heads at the same time [28,29]. In recent years, with the improvement of people’s living standards in China, the increase in demand for meat, eggs and milk has promoted the rapid development of animal husbandry, and the demand for feed has increased significantly, which has increasingly highlighted the contradiction between grain and feed in China. In fact, there is an objective requirement to change from a two-harvest cultivation mode of RR to a production mode of RR that can be used for both grain and feed. Our research team has been conducting research in this field for a long time and has achieved certain results [30]. This method of producing grass from current-season rice and harvesting grains from subsequent-season rice has been successful in the southern rice growing-areas of China in recent years. This approach has been implemented widely in the United States [31], Australia [32], and Japan [33] and other developed countries and regions have widely implemented it. At present, crops that can be used for both grain and feed purposes mainly include major cereal crops such as rice [29] and barley [31]. Previous studies have also investigated the response of forage biomass to cultivation measures such as the cutting period, stake height, planting density, and nitrogen fertilizer application in a “double harvest” feed rice model that mainly harvests two seasons of forage [34]. At present, the exogenous Se that has been extensively studied and reported on is mainly inorganic selenate or selenate; however, relatively few reports on organic Se and nano-Se in rice exist, and the effects of exogenous Se application in RR are still unclear. Hu et al. (2018) [35] studied the absorption and transport of nano-Se in wheat seedlings via hydroponic technology, and Wang et al. (2017) [36] reported that the foliar application of nano-Se to wheat efficiently increased the grain Se concentration, and that Se enrichment with nano-Se was inferior to enrichment with selenite, selenate, or selenomethionine (SeMet). Lyu et al. (2022) [37] reported the size-dependent transformation, uptake, and transportation of SeNPs in a wheat–soil system; Yuan et al. (2023) [15] reported that the foliar application of organic Se increased the Se concentration in rice. To date, existing research has focused mostly on the absorption and/or transformation of Se in rice or wheat in a controlled hydroponic system or in the field, but there are relatively few studies that have focused on the effects of different forms of Se in RR.

The purpose of our study was to identify the Se-enriched mechanism of RR in Se-enriched rice production and to determine whether the interaction of Se concentration with the different forms of Se in Se-enriched rice followed these two growth stages. Different types of exogenous Se could more effectively accumulate in various parts of the rice plant and improve and enhance the nutritional value of RR plants. The findings of this study will help us select appropriate forms and concentrations of Se in the Se-enriched RR-producing areas and establish high-quality and efficient strategies for planting crops in RR-producing regions.

## 2. Materials and Methods

### 2.1. Plant Material

The Chinese-bred ‘Liangyou 6326’ (LY) hybrid rice cultivar was chosen for our experiments, with the parental origin ‘Xuan 69S’ × ‘WH 26’ and the national certification number 2007013, was chosen for our experiments. LY is an indica type of two-line hybrid rice, and its entire reproductive period is 130 days. Seeds were obtained from the Collaborative Innovation Center of Henan Grain Crops, Henan Agricultural University. The initial germination and seed moisture contents were >95 and 10.12% (dry weight basis), respectively. All experimental plant research complied with the IUCN Policy Statement on Research Involving Species at Risk of Extinction and the Convention on the Trade in Endangered Species of Wild Fauna and Flora.

### 2.2. Soil Characteristics and Experimental Design

#### 2.2.1. Experimental Site and Growth Conditions

Fresh paddy soil (yellow-brown soil) was collected from a rice field within a 0–20 cm depth soil cultivation layer using the five-point method in Lilou Village, Guangshan County, Henan Province (112°58′–113°30′ N, 27°54′–28°22′ E). The soil contained 38.3 g·kg^−1^ of organic matter, 2.97 g·kg^−1^ of N, 7.2 mg·kg^−1^ of available phosphorus, 153 mg·kg^−1^ of rapidly available potassium, and 0.43 mg∙kg^−1^ of Se, and the soil pH was 5.8.

This experiment is a field experiment on the entire growth period of rice. Please refer to the method proposed by Peng et al. (2020) [30] for specific details. The experimental RR leaves 35 cm of stubble at the heading and flowering stages of the current season, cuts and harvests the forage, dries it as dry feed, and partially uses it to test forage indicators. RR represents the number of harvested rice grains at maturity.

In the current season of rice, pure nitrogen was applied at a rate of 240 kg·hm^−2^, with a base fertilizer/tiller fertilizer/panicle fertilizer ratio of 4:2:4. The base fertilizer was applied 1 day before transplanting, the tiller fertilizer was applied 7 days after transplanting, and the panicle fertilizer was applied during the four-leaf period. With the ratio of N:P_2_O_5_:K_2_O = 2:1:2, phosphate fertilizer was applied as the base fertilizer once, while potassium fertilizer was applied at 50% as the base fertilizer and 50% during the four-leaf period. Sow on the 25th of March (2021), a machine was transplanted on the 18th of April, with a plant spacing of 14 cm and a row spacing of 28 cm. The RR in the subsequent season was treated with 150 kg·hm^−2^ of pure nitrogen and 90 kg·hm^−2^ of K_2_O on the same day after harvesting. The field experimental plot covers an area of 15 m^2^, with 10 plots for each treatment and control, which was repeated 3 times. Before the experiment, the selected fields were marked and divided into zones, with a 3 m protective row in each zone, totaling 30 plots. All the plots were randomly distributed, with each treatment area being 3 × 5 or 15 m^2^.

The treatment methods used for the experiments included foliar spraying and three forms of Se. Sodium selenite (SS, Na_2_SeO_3_, analytical pure grade) and selenomethionine (SeMet, analytical pure grade) were obtained from Nanjing Chemical Reagent Co., Ltd., Nanjing, China, and nano Se (NS) was obtained from Professor Pan Canping from China Agricultural University (which was synthesized through microbial fermentation with selenite as the material). The average particle size of the nano-Se was 15.886 nm. It is a purple-red powder that is easily soluble in water [38,39]. Three Se forms were used as the sources of Se because they are ambiguous and are more efficient than other form of Se sources in RR production. Different concentrations of Se were chosen on the basis of early findings [40], so this experiment set three different treatment concentrations for each type of Se source, labeled as follows:

T0: the control treatment (CK, only distilled water only); T1-SS at a low rate (50 mg·L^−1^ of SS), T2-SS at a medium rate (100 mg·L^−1^ of SS), and T3-SS at a high rate (150 mg·L^−1^ of SS); T1-SeMet at a low rate (50 mg·L^−1^ of SeMet), T2-SeMet at a medium rate (100 mg·L^−1^ of SeMet), and T3-SeMet at a high rate (150 mg·L^−1^ of SeMet); and T1-NS at a low rate (150 μg·L^−1^ of NS), T2-NS at a medium rate (450 μg·L^−1^ of NS), and T3-NS at a high rate (900 μg·L^−1^ of NS).

When applied in the field, a Tween-20 (Gaidechem Co., Ltd., Shanghai, China) activator was added to the working solution, and the applied concentration was 0.05%. One liter of solution was used per plot each time, which was repeated 3 times, and a total of 6 L of liquid was sprayed over 2 seasons. There were approximately 1500 seedlings in each plot.

Se sources were evenly sprayed on the upper leaf surfaces once every two days at the rice booting stage from 24 June 2021 to 3 July 2021. Between the tillering and early booting stages, leaves of the the current-season rice were sprayed in the leaves of each plot every two days for three consecutive treatments, whereas the blank control was not be treated with Se and sprayed with clean water. The same operation was repeated once during the subsequent rice season, from 1 September 2021 to 8 September 2021.

Straw was harvested during the early booting stage (25 July 2021) after Se treatment, with a stubble height of 35 cm. Afterwards, daily management followed the working system of the farm field.

#### 2.2.2. Determination of Morphological Indicators and Se Contents in Grains

During harvesting, shoot height, panicle length, kernels per spike and 1000-grain weight were recorded. The plant height was measured from the soil surface to the highest upright rice position and was recorded in meters. Panicle length refers to the length of the entire rice panicle after being spread flat.

### 2.3. Measurement Items and Methods

#### 2.3.1. Sampling and Measurement Times for Physiological Indicators

Some physiological indicators were measured after Se treatment during the booting stage of the current season rice (first stage enzyme activity) and before rice was cut (second stage), and the plant growth status and yield indicators were determined based on the results of the subsequent rice measurement (third stage enzyme activity). The total biomass and yield indicators of the RR were determined based on the results of the subsequent season of rice cultivation (enzyme activity stage 3). Photosynthesis was measured two weeks after Se spraying in the subsequent season. After the straw was cut, it was weighed and air-dried, and three intact leaves from the upper part were taken as samples to determine each nutrient element. The above operation was repeated before the tillering stage of the subsequent rice season, and photosynthesis and enzyme activity were measured in the third stage. The total Se and other nutrient contents, as well as the plant dry matter weight, fresh matter weight, thousand-grain weight, and total grain weight (yield), of the above-ground parts of mature rice grains were measured.

#### 2.3.2. Measurement of Rice Plant Biomass and Yield

The plant height and spike length were measured via a ruler, whereas the dry weight, fresh weight, and 100-grain weight were measured via an analytical balance. The seedling plant height was determined with a ruler to measure the natural height from the base of the stem to the longest leaf before the tillering stage, and the adult plant height was determined before harvest.

The current-season rice was harvested in the early stage of booting, dried and weighed, and the entire plant of the subsequent-season rice was also weighed; the sum of the two parts was taken as the total biomass. Fresh samples of whole plants subjected to different treatments were weighed and recorded. Then, the fresh grains were air-dried and the 1000-grain weight was determined. The dry weights of the different treatments were then recorded.

#### 2.3.3. Chlorophyll Content

A SPAD chlorophyll meter (SPAD-502; Konica, Minolta Sensing, Inc., Tokyo, Japan) was used to determine the chlorophyll value.

#### 2.3.4. Determination of Quality Indicators for Forage and Rice

The determination of quality indicators for forage was conducted as described by Peng et al. (2020) [30] after the current season rice was harvested in the early stage of booting and air-dried according to the National Standard “Evaluation of forage nutritional quality—Grading index (GI) method (GB/T23387-2009)” to determine various indicators [41].

The rice samples were collected, washed with deionized water, and then oven-dried at 55 °C. The dried samples were ground and filtered through a 1 mm nylon sieve for nutrient analysis. For the Se assays, the concentration of Se in the solution was determined via an 8200 atomic fluorescence spectrometer (JITIAN Company, Xiamen, China) (GB/T 21729, 2008) [41] as described by Zhang et al. (2001) [42]. A certified reference material (Bush Twigs and Leaves, GBW07603/GSV-2) was used as the quality control sample to calculate the recovery rate of Se (96.5%) (https://www.bwsm.org/ 3 February 2022).

#### 2.3.5. Determination of Se Content and Se Speciation

The Se speciation assay in plants was conducted as described by Yin et al. (2019) [17]. For total Se content determination, the dried leaf or grain samples were ground and digested in a mixture of HNO_3_-HClO_4_ (4:1), and the concentration of Se was measured via inductively coupled plasma atomic emission spectroscopy (ICP-AES) (SPS 1200 VR, Seiko Co., Ltd., Tokyo, Japan). Furthermore, water-extractable Se was extracted using deionized water, as described previously (Zhang et al. 2001) [42]. Afterwards, the entire operation process was carried out as described earlier [39].

#### 2.3.6. Digestion of Samples and Determination of Elemental Concentrations

After preprocessing, approximately 0.1 g of shoot or grain sample was digested following the methods of Zhu et al. (2020) [43]. The concentrations of Fe, Zn, Cu and Mn were determined via inductively coupled plasma–mass spectrometry (ICP-MS, Nexlon 300X, Shelton, CT, USA). The standard reference material (shrub leaves, GBW07603, GSV-2) and blanks were used to ensure the accuracy of the elemental analysis. The reference material was purchased from the China Standard Material Center, and the determination recovery was 94–106%.

#### 2.3.7. Photosynthetic Rate Measurement

The net photosynthetic rate (Pn), transpiration rate (Tr), intercellular CO_2_ concentration (Ci), and stomatal conductance (Gs) of the second fully unfolded leaf were measured between 8:00 and 18:00 on a sunny day using an open-flow infrared gas analyzer adapted with light and temperature control systems (LiCOR 6400, Li-COR, Lincoln, NE, USA) as described previously (Ahammed et al., 2018) [44]. The following describes the conditions used for the estimation of gas exchange attributes: the leaf chamber volume gas flow rate was 288 mL·min^−1^, the molar air flow per unit leaf area was 390 mmol·m^−2^·s^−1^, the atmospheric pressure was 90.4 kPa, the water vapor pressure in the chamber was 6.3–7.5 mbar, the leaf temperature was 24.5 °C and the air CO_2_ concentration was 379 μmol·mol^−1^. Measurements were taken 2 weeks after the last Se treatment (25 September 2021) on three plants from each of the three biological replicates for each treatment.

#### 2.3.8. Activity of Enzymes Correlated with Antioxidant Enzyme System

Kits from Biotechnology Co., Ltd. (KeMing), Suzhou, China, were used to determine the activity of enzymes, including superoxide dismutase (SOD, SOD-2-Y), peroxidase (POD, POD-2-Y), catalase (CAT, CAT-2-Y), glutathione (GR, GR-2-W), glutathione peroxidase(GSH-Px, GSH-2-W) and GSSG (GSSG-2-W) in rice leaves. The measurement procedures were carried out according to the kit instructions. The extraction mixture was composed of fresh leaf plus extracts [the ratio of leaf mass (g)/extract volume (mL) was 1:5]. The mixture was then ground to a homogenate in an ice bath. The homogenate was centrifuged (4 °C) at 8000× *g* for 10 min. The supernatant was then collected and placed on ice for the determination of different enzyme activities. The supernatant was mixed with different reagents (supplied by the company) and incubated in a water bath. The supernatant was used for assays of the antioxidant enzyme activities by a microplate reader [45]. Sampling was conducted three times: twice in the current season and once in the subsequent season (18 July; 25 July; 25 September 2021).

#### 2.3.9. Determination of the Characteristics of the Grain Quality

The methods for determining the whole grain percentage and chalkiness were performed according to the National Standard of the People’s Republic of China “GB/T 17891—1999 High Quality Rice” (https://www.antpedia.com/standard/406025-1.html; 12 March 2022). The spectral viscosity characteristics of the rice flour were determined via a Super3 7VA starch viscosity rapid analyzer produced by New Port Scientific Instrument Company, Brisbane, Australia, and were determined via AACC procedures according to Vasanthem. T. et al. [46].

To determine the amylose content (AC), gelatinization consistency (GC), gelatinization temperature(GT), and alkali spreading value (ASV) of the rice, the powdered samples were scanned using the Near-Infrared Grain Analyzer Perten DA7250, Stockholm, Sweden. According to the accuracy requirements, the average value for the samples was calculated if the ratio of the difference between the two tests resulted in an average value of less than 2%. The amino acid (AA) content assay kit (AKAM001M) and DPPH free radical scavenging capacity assay kit (A153-1-1) were purchased from the Boxbio (Beijing, China) and Hangzhou Acon (Hangzhou, China), respectively. If the requirements were not met, the samples were retested, and the average value was calculated.

#### 2.3.10. Determination of Starch, Protein and Amino Acid

Rice grain powder was extracted using a 80:20 (*v*/*v*) ethanol/water mixture to determine the starch content, and the content of the extract was determined colorimetrically following the modified phenol–sulfuric acid method of Buysse and Merckx (1993) [47]. The determination of resistant starch is followed methods described previously [48]. The determination of protein content refers to “NY/T 3-1982, Method for detemination of crude protein in cereals and bean seeds (semi-micro kjeldahl method)” (https://max.book118.com/html/2019/1118/5112212011002202.shtm; 3 May 2022) and is determined via the semimicro Kjeldahl method with a sample dosage of 0.1 g sample of refined rice flour. The amino acid content was determined via an amino acid analyzer (Hitachi L-8900 amino acid analyzer, Hitachi, Tokyo, Japan).

### 2.4. Statistical Analysis

Significant differences in the measured traits were detected by ANOVA procedures in the JMP V12.0 statistical software from SAS (version 9, SAS Institute, Inc., Cary, NC, USA). The data are expressed as the mean values ± SDs. Significant differences between different Se application levels were detected via Fisher’s protected LSD test at α= 0.01 and α = 0.05. The methods of correlation analysis and principal component analysis (PCA) can be found in [49].

## 3. Results

### 3.1. Morphological Attributes

The height of seedlings and adult plants, the spike length, and the 1000-grains weight of RR were strongly affected by the supplementation with Se. Compared with the control, application of 100 mg·L^−1^ (T2-SS) of Se significantly increased the seedling and adult plant height by 16.65% and 10.28%, the chlorophyll value by 18.60%, and the spike length and 1000-grain weight by 4.93% and 15.52%, respectively, in LY (Table 1). However, a relatively high concentration of Se (150 mg·kg^−1^) negatively influenced the growth traits, with the highest concentration combination of the three types of exogenous Se triggering some reductions in 1000-grain weight, with T3-SeMet reduced by 6.90%, and T3-NS by 9.20%, respectively, compared with those of the control plants. The chlorophyll content (SPAD value) was significantly increased in response to the low-Se treatment with three exogenous Se leaf sprays, and the lower concentration of Se increased the most SPAD value the most by 19.41%, 24.1% and 22.01%, respectively, compared with that of the control plants (Table 1). However, as the concentration of Se increased, the observed value actually decreased. Given that yield indicators are based on thousand-grain weight, various changes occurred. Although there were differences in the performance of various exogenous Se sources, the growth of rice did not increase but rather decreased with increasing Se application concentration (>100 mg·L^−1^), indicating that although high-Se treatment is beneficial for seedling growth, it is not conducive to the later development of rice.

### 3.2. Se-Content of Different Parts of RR

The Se content in different parts of RR without Se application tended to decrease from the roots to the above-ground parts as follows: roots > stems > spike stalk > grains. The Se content in the grains was very low, at only 0.043 mg·kg^−1^. The exogenous application of Se increased the content of Se in shoots and roots, with the highest content increasing 20-fold (Table 2). The effects of the three Se sources on the Se content in various parts of RR, whether in stems, leaves, spike stalk, or grains were in the order of T2-SS > T2-NS > T2-Semet. However, the distributions of the three Se sources in various parts still followed the aforementioned pattern, with the highest content occurring in the roots. Under different concentrations of the same Se source, the changes in the contents of individual parts tended to increase with increasing Se concentration, followed by a decreasing trend. The highest Se content of each Se source occurred in the combined medium-concentration treatment, but there were still differences among the three Se sources. For example, for the above-ground stems and leaves harvested in the early stage, the Se concentration of the T2-SS combination was 0.929 mg·kg^−1^, whereas the high-concentration treatment combinations of the other two Se sources were comparable, but presented slightly lower concentrations, 0.461 mg·kg^−1^ and 0.684 mg·kg^−1^, respectively, which were reduced by 50.38% and 26.37%. In terms of the Se content of the grain in the subsequent season, the T2-SS combination was still the best, with the highest Se content. However, as the treatment concentration increased, the Se content in the grains actually decreased. For both the organic Se and nano-Se treatments, the highest Se content of the grains occurred in the high-concentration treatment combination, but it was still lower than the 0.757 mg·kg^−1^ Se content of the T2-SS combination, with reductions of 46.90% and 49.54%, respectively.

Different types and concentrations of Se application affect the total Se concentration in leaves and brown rice. When the concentration of Se applied increased, the accumulation of Se in brown rice increased, but as the concentration of Se sprayed further increased, the Se concentration in brown rice decreased. Among the three exogenous Se treatments, seven combinations exceeded 200 μg·kg^−1^, meeting the standard for Se-rich rice (the control group had a Se concentration of 43 μg·kg^−1^ in brown rice) (Supplementary Tables S1).

Figure S1 also shows that there was little difference in the contents of the stems, leaves, and grains treated with sodium selenite, whereas there was a significant difference in the contents between the two organs treated with T-SeMet and those treated with T-NS. These findings suggest that RR has different mechanisms for absorbing and transporting these three forms of Se, and that inorganic Se treatment is beneficial for Se enrichment in grains.

### 3.3. Nutritional Quality and Trace Element Content of RR Stems and Leaves

#### 3.3.1. Nutritional Quality of RR Stems and Leaves

The effects of Se application on the quality of RR straw in this experiment are shown in Table 3. The national standard for the quality index of crude protein was 7.8%. Among the 10 treatment combinations in this experiment, 6 treatment combinations reached or even exceeded this index. The percentage increase in the T3-SS combination compared with the control was 17.83%, which was 0.8 greater than the national standard. Only the T2-NS and T3-NS treatment indicators were lower than those of the control, with reductions in percentage of 2.47 and 1.93, respectively. For another indicator of starch, the optimal treatment combination was T2-SS, with the starch content increasing by 2.43%.

#### 3.3.2. Trace Element Content of RR Stems and Leaves

Table 4 shows that the application of Se has a certain effect on the contents of trace elements in rice straw. The combinations that had a significant effect on iron content included T1-SS, T2-SS, T2-NS, and T1-SeMet, whose contents increased by 67.5%, 58.02%, and 41.33%, respectively, compared with those of the control. All the combined effects of the treatments were greater than those of the control, and significant differences were observed between the treatments. Compared with the control, the combination of T1-NS and T2-SS had better effects, increasing the copper content by 7.26% and 3.9% respectively, but the difference was not significant. The effects of the treatments, including T2-SeMet, T3-SeMet, and the T2-NS combination, were smaller than those of the control.

Similar to the effects of the iron treatment, the effects of all the combination treatments were greater than those of the control, and significant differences were observed between each treatment. The three combinations with the greatest increases were T3-SS, T2-SS, and T1-NS, whose effect values increased by 65.2%, 61.7%, and 51.2%, respectively, compared with those of the control. There was a significant change in the treatment effect for manganese, with five combination effects increasing and four combination effects decreasing. The top three combinations with the largest increase were T1-SS, T3-SS, and T1-Semet, with corresponding increases of 29.59%, 32.12%, and 20.72%, respectively.

### 3.4. Photosynthetic Indicators of RR Leaves

#### 3.4.1. Photosynthetic Indicators of RR Leaves in Current Season

First, the Pn values of the five combinations significantly increased compared with those of the control group; in particular, the T2-SS and T3-SS combinations had better effects, increasing the Pn values by 20.4% and 13.05%, respectively. The Pn of the T1-SeMet and T1-SS combinations slightly increased, but the difference from that of the control group still reached a significant level (Table 5). In addition to an increase in the Pn, the T2-SS treatment combination resulted in a significant decrease in the levels of Tr and Ci compared with those of the control, whereas Gs increased. In addition to a decrease in Pn, the T3-SeMet combination resulted in an increase or decrease in the other three indicators; notably, in all the treatment combinations, except for T2-SeMet, the Tr significantly decreased. Compared with those of the control, the combinations with the greatest reductions were T3-NS, T2-NS, and T3-SeMet, which decreased by 65.71%, 48.57%, and 40.0%, respectively. The combinations with increased Gs included the T1-SS, T2-SS, and T1-SeMet combinations.

Compared with the control, multiple treatment combinations significantly increased the Pn of the RR in the subsequent season. The order of Se sources with significant efficiency enhancement was T-SS >T-NS >T-SeMe, and the T1-SS, T2-SS, and T3-SeMet combinations increased by 26.44%, 18.86%, and 25.97%, respectively, compared with those of the control group.

#### 3.4.2. Photosynthetic Indicators of RR Leaves in Subsequent Seasons

The Ci of the multiple treatment groups decreased significantly, and the combinations with the greatest decrease were T1-SS (54.25%), T3-SeMet (50.65%), and T1-SeMet (44.11%) (Table 6). The effects of various Se treatment combinations on the Tr and Gs seem to be unclear, with varying degrees of variability.

### 3.5. Antioxidant Enzyme Activities

**Figure 1:**
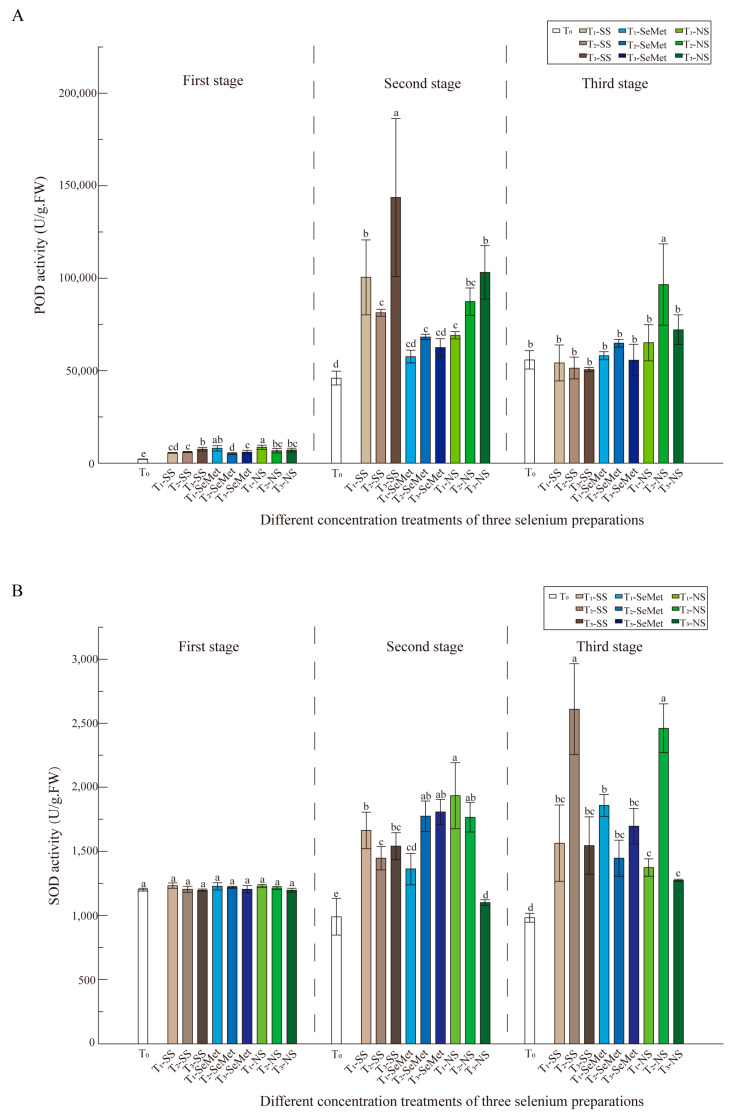
The effects of three exogenous Se sources on the antioxidant enzyme system of ‘LY’ RR after leaf spraying treatment. (**A**) The activity of POD; (**B**) the activity of SOD. Data are presented as means ± standard deviations. For the different Se doses of the same Se form, values not displaying the same letter are significantly different (*p* < 0.05). The Figure 2 and Figure 3 are the same.

As shown in Figure 1A, the POD activity of LY in the first stage was lower than that in the other two stages; POD activity decreased significantly with increasing sodium selenite concentration but then increased again, and the high-concentration combination of sodium selenite resulted in a 2-fold increase in POD activity compared with that of the control. During the third stage, the enzyme activity of the different Se sources gradually increased, with the highest concentration combination of nanoselenium being 150% that of the control. As shown in Figure 1, POD activity was significantly affected by stages, and significantly affected by the treatment concentration. SOD activity (Figure 1B) was greater in the third stages than in the other stages, and the differences in enzyme activity among the multiple treatments in the first and second stages were relatively small, whereas the differences increased significantly in the third stage. Compared with the control, the combination of medium-concentration sodium selenite and nano-Se resulted in a significant increase in activity with a magnitude over 2 times higher (Figure 1B).

**Figure 2:**
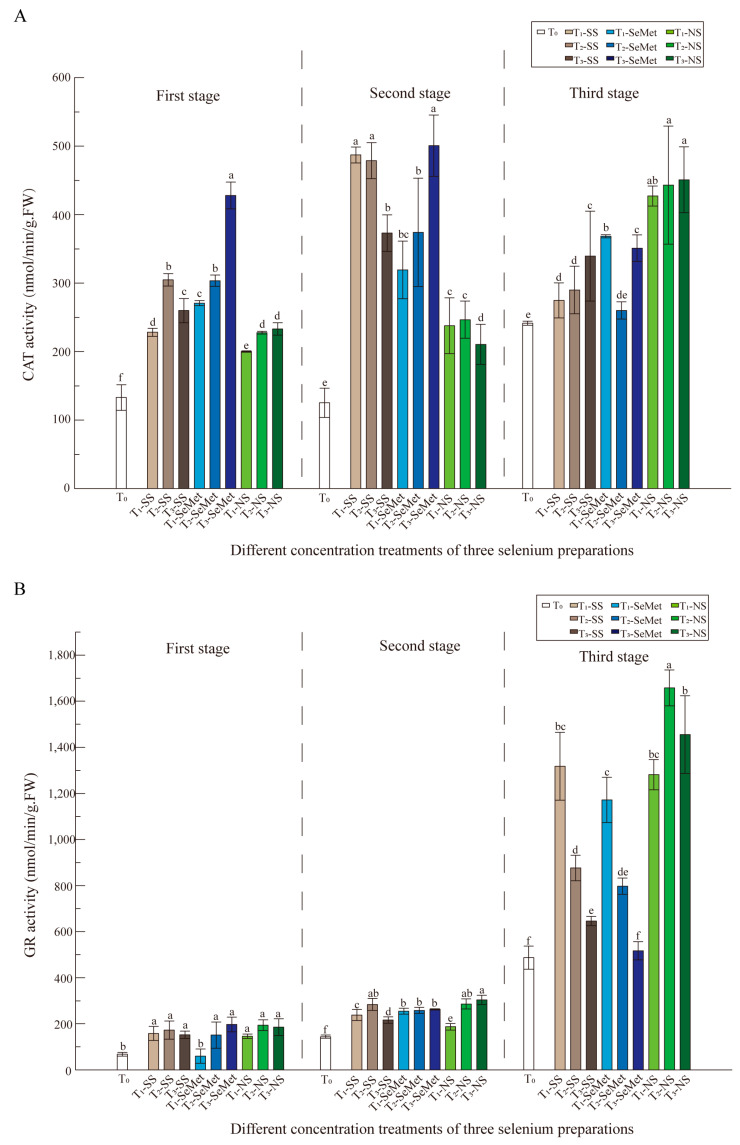
The effects of three exogenous Se sources on the antioxidant enzyme system of ‘LY’ RR after leaf spraying treatment. (**A**) The activity of CAT; (**B**) the activity of GR.

The increasing trends in CAT activity are shown in Figure 2A for the three Se forms with increasing treatment concentrations, but the decrease was more pronounced with increasing developmental stage (second stage) in the T-SS and T-NS treatments. As shown in Figure 2A, CAT activity was significantly affected by the treatment concentration and the different Se forms.

The GR activity (Figure 2B) varied slightly between the first and second periods, and although there were significant differences among the treatment combinations, there seemed to be no significant differences among the selenium sources. There was a significant increase in enzyme activity during the third period, and the combination of T-SS and T-SeMet resulted in a similar pattern of decreasing activity with increasing concentration. The average enzyme activity level under the nano-Se treatment was relatively high, but there was a trend toward low–high–low activity at the three treatment concentrations, indicating that low or medium concentrations of the three Se sources seem to be beneficial for promoting GR enzyme activity.

**Figure 3:**
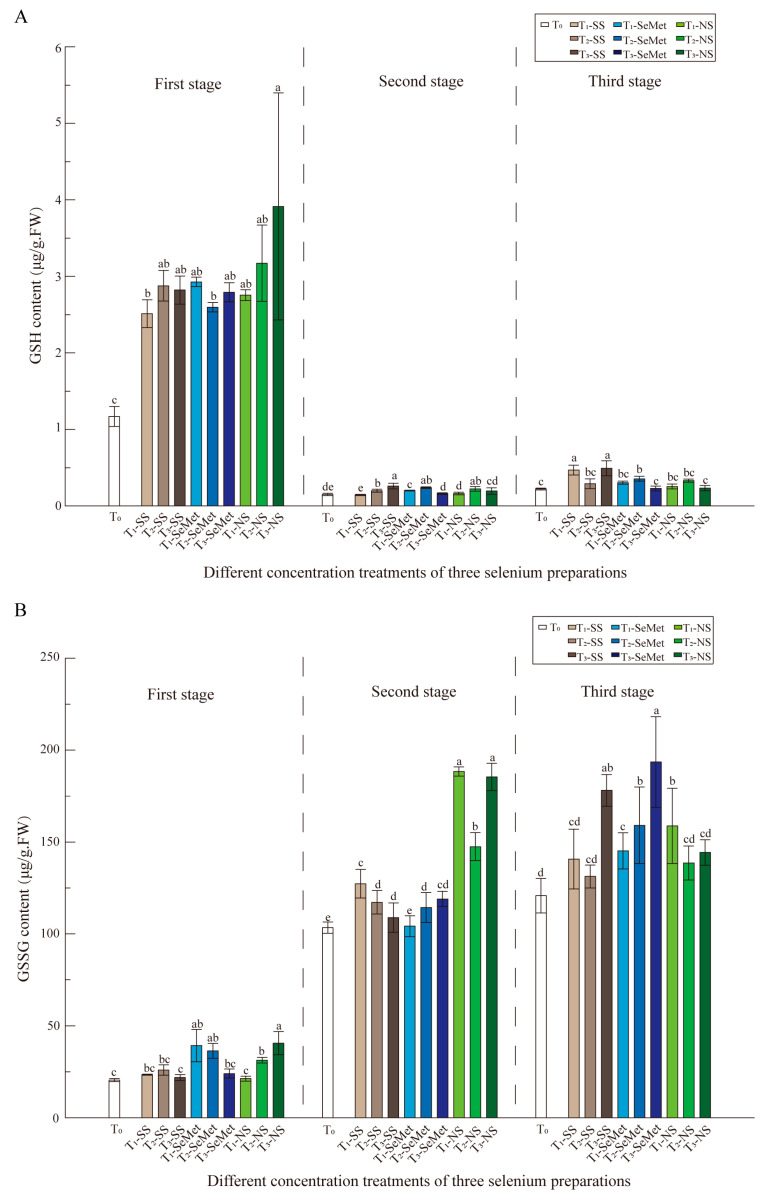
The effects of three exogenous Se sources on the antioxidant enzyme system of the ‘LY’ RR after leaf spraying treatment. (**A**) The content of GSH-Px; (**B**) the content of GSSG.

The GSH-Px content (Figure 3A) was significantly affected in the first stage, and the peptide content of the nine Se treatment combinations was approximately twice that of the control, with the most significant effect observed in the high-concentration T-SN treatment combination, which was 3.1 times greater than that of the control. The content of each treatment in the second and third stages decreased significantly to less than one-third of that in the first stage, and there were still significant differences among the three Se sources.

The contents of the three stages tended to be low–high–high, and the contents of the second and third stages increased significantly compared with those of the first stage. The performance of different Se sources in the second stage varied: the effect of T-SS treatment decreased with increasing Se concentration, whereas the effect of T-SeMet treatment increased with increasing Se concentration. Compared with that of the control, the amplification effect of these two Se sources was smaller. During the third stage, the overall content continued to increase, and all three Se sources promoted an increase in the GSSG content (Figure 3B). Therefore, the impacts of different concentrations of Se within and between three different Se forms were significant for all physio-biochemical attributes.

### 3.6. Grain Yield and Total Biomass

The average biomass of the RR in the subsequent season and the grain yield with no Se application were 17.21 and 6.94 t·ha^−1^. The experimental results revealed that the LY variety presented different responses to the three Se sources. Compared with the CK treatment, foliar spraying of T-SS at the growth stage had a significantly positive effect on grain yield and total biomass, with an increase in total biomass and average grain yield of 2.09% and 14.55%, respectively. The experimental results also revealed that the LY variety had different responses to the three Se sources and that the effect of organic Se on yield was unstable, whereas nano-Se had certain side effects on total biomass. The interaction effect between Se species and Se application concentration can be observed in the total biomass (Table 7).

### 3.7. Se Concentration and Accumulation Distribution Ratio in RR Grains and Leaves

The Se source had a significant effect on the concentrations of Se in different parts of rice. Table 8 shows that the organic Se and protein Se contents in brown rice significantly increased, and the combination of T2-SS and T3-SS resulted in the greatest increase in organic Se, with contents of 0.817 and 0.575 mg kg^−1^, respectively. The combination of T3-NS was 0.367 mg kg^−1^, the organic Se ratio was 90.62, and the corresponding protein Se content was 0.328, which was 11.6 times greater than that of the control. The detection results for organic Se were all above 85%, and there were significant differences between the different Se source treatments.

**Figure 4:**
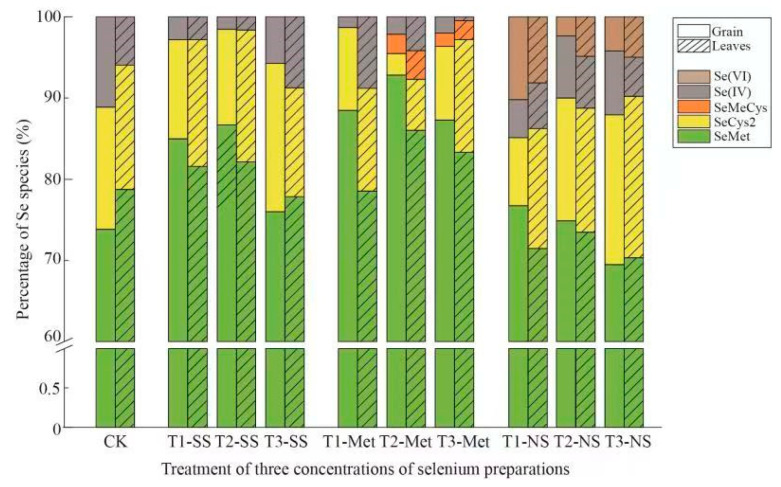
The percentages of Se species in the leaves and grains of RR resulting from foliar spraying of exogenous Se. Note: There are percentages of Se speciation from the leaves and grains of RR resulting from the foliar spraying of exogenous Se. Figure 4 shows the results in Table S1.

Notably, when the Se source was organic Se, the content of organic Se in the rice grains and leaves was high, and the type of protein Se changed. SeMeCys was present only in the combination of organic Se treatment (T-SeMet), and the content of SeMeCys in the leaves and grains was 3.52% and 2.39%, respectively, while the protein Se content was the lowest in the combination of T-NS treatment (Figure 4).

### 3.8. Brown Rice Quality of RR

#### 3.8.1. Trace Element Contents

The effects of Se application on the trace element contents of RR brown rice in this study are shown in Table 9. Compared with the control, Se application significantly increased the zinc content in brown rice; three combinations resulted in the greatest increase with increases of 31.15% (T2-NS), 22.22% (T3-NS), and 20.82% (T1-NS), and the difference in the zinc content between each combination of brown rice and the control reached a significant level. The content of iron in brown rice greatly increased, and the three combinations with the greatest increase were T2-SS, T3-SS, and T3-SeMet, with increases of 33.33%, 32.66%, and 28.28%, respectively, with significant differences. Compared with the control, only T2-SeMet significantly increased the copper content, with an increase of 3.42%, while most other combinations resulted in a decrease, and the difference was also significant. Compared with those of the control, the manganese contents of T2-SS and T3-SS significantly differed, with increases of 11.2% and 9.37%, respectively. The other treatment combinations significantly reduced the manganese content, with the top three treatments resulting in the greatest reductions of 38.42%, 25.99%, and 15.38%, respectively (Table 9).

#### 3.8.2. The Processing and Appearance Quality of Brown Rice

The effects of Se application on the processing and appearance of RR brown rice in this study are shown in Table 10. Compared with those of the control, the overall brown rice percentage, polished rice percentage, and whole polished rice percentage of each treatment were comparable. The variation range of the brown rice percentage in the T-SS treatment ranged from 81.8%~82.8%, the variation range of the polished rice percentage ranged from 74.9%~75.1%, and the variation range of the whole polished rice percentage ranged from 41.9%~64.4%. The variation range of the brown rice percentage in the T-SeMet treatment ranged from 81.6%~83.0%, the variation range of the polished rice percentage ranged from 73.9%~75.5%, and the variation range of the whole polished rice percentage ranged from 47.8%~48.0%. The variation range of the brown rice percentage in the T-NS treatment ranged from 82.1%~83.2%, the variation range of the polished rice percentage ranged from 74.5%~77.0%, and the variation range of the whole polished rice percentage ranged from 50.3%~61.8%. Compared with the brown rice percentage and polished rice percentage, the variation range of the whole polished rice percentage was greater (Table 10).

As shown in Table 10, different exogenous Se treatments significantly affected the processing quality and appearance quality of RR grains, and the differences between the treatments were significant. The brown rice percentage of the T-SS combination at the three treatment levels decreased significantly, by 1.6%; the overall rice percentage index was 65.9% in the control group, while that of the T2-SS treatment combination was 68.4%, which was 3.79% higher than that of the control group. Most combinations of different Se source treatments reduced the chalkiness rate, chalkiness size, and degree of chalkiness, with only a few combinations showing an increase. The differences between the treatments were significant.

#### 3.8.3. The Cooking and Eating Quality

The Se source had significant effects on the cooking and eating quality of rice. Table 11 shows that the RS values of brown rice significantly changed, with only the T3-NS treatment combination having lower indicators than the control, while all the other combinations presented an increase. The three combinations with the highest growth rates were T2-SS, T3-SS, and T3-SeMet, which increased by 435.90%, 358.97%, and 253.85%, respectively, compared with those of the control group. After treatment with exogenous Se, the AC values of brown rice increased significantly. The treatment combinations with the greatest increases were T1-SS, T2-SS, and T1-SeMet, which increased from 14.80% in the control to 24.12%, an increase of 62.97%. The T2-SS combination followed closely behind the other combinations, increasing by 54.80% compared with that of the control. In terms of another antioxidant activity index, exogenous Se treatment improved the antioxidant capacity of most combinations, with the T1-SS combination resulting in the greatest improvement in the antioxidant capacity index, increasing by 20.25%, followed by the T1-SeMet treatment, which resulted in an increase of 15.21%. Compared with the control, the four combinations had lower antioxidant activity indicators.

### 3.9. Correlation Analysis

**Figure 5:**
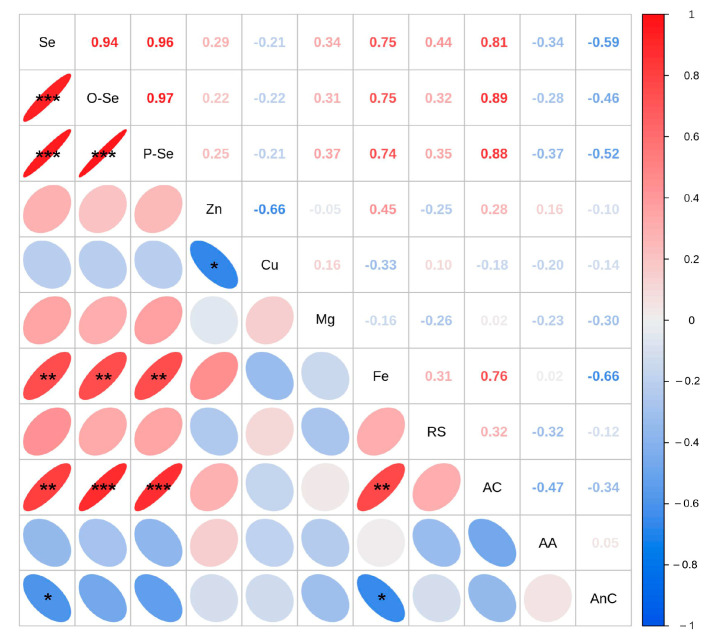
Correlation analysis of 11 nutritional qualities. *, **, and *** denote significance levels of 0.05, 0.01, and 0.001, respectively. Note: O-Se—organic Se; P-Se—protein Se; RS—resistant starch; AC—amylose content; AA—amino acid; AnC—antioxidant activity.

A correlation analysis of the 11 nutritional quality indicators revealed significant relationships among these parameters (Figure 5). Se was positively correlated with organic Se, protein Se (*p* < 0.001), Fe (*p* < 0.01), and the amylose content ((*p* < 0.01), but negatively correlated with Zn and Cu (*p* < 0.05), Se with antioxidant activity, and Fe (*p* < 0.05). The amylose content was positively correlated with the organic Se (*p* < 0.001), protein Se (*p* < 0.001), and Fe (*p* < 0.01) contents.

### 3.10. Principal Component Analysis

**Figure 6:**
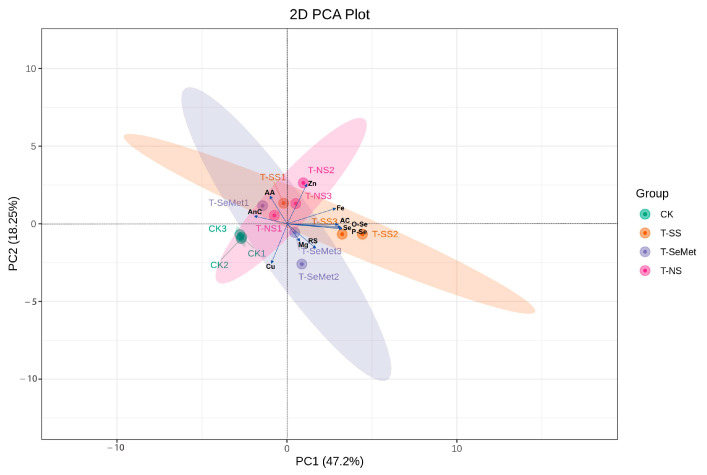
PCA of quality indicators for three Se forms at three Se doses in RR. Note: T0: the control treatment (CK, distilled water only); T-SS1 at a low rate (50 mg·L^−1^ of SS), T-SS2 at a medium rate (100 mg·L^−1^ of SS), and T-SS3 at a high rate (150 mg·L^−1^ of SS); T-SeMet 1 at a low rate (50 mg·L^−1^ of SeMet), T-SeMet 2 at a medium rate (100 mg·L^−1^ of SeMet), and T-SeMet 3 at a high rate (150 mg·L^−1^ of SeMet); and T-NS1 at a low rate (150 μg·L^−1^ of NS), T-NS2 at a medium rate (450 μg·L^−1^ of NS), and T-NS3 at a high rate (900 μg·L^−1^ of NS).

Principal component analysis was conducted on the quality indices of the three Se forms with three Se doses in 2021 (Figure 6). Four principal components with eigenvalues > one were extracted. The cumulative contribution rate of the first two principal components was 65.45%, indicating high representativeness (Supplementary Tables S2 and S3). PC1 and PC2 account for 47.20% and 18.25% of the variation, respectively, with small differences within each sample group. The data are reliable and reproducible. The first two principal components (PC1 and PC2) were utilized to create a score-load diagram. Different Se forms were distinguished on either side of the PC1 axis.

Figure 5 shows that, compared with those in the control group, the contents of Zn, Fe, AC, Se, O-Se, P-Se, RS, and Mg in each treatment group were greater, especially in the T-SS treatment.

## 4. Discussion

To achieve sustainable development, people must produce sufficient food of guaranteed quality, and the use of Se-enriched rice production technology to produce high-quality rice with functional components is currently an important research area [21,25,30]. The use of grain-and-feed dual-use technology in the foliar application of Se in the RR production system is an important cost-effective approach.

### 4.1. The Exogenous Application of Se Promoted the Appearance Quality and Photosynthetic Traits of RR

The production of Se-enriched rice is primarily aimed at increasing the Se content in the grains, because exogenous Se application can significantly increase the Se content of crops, and applying Se fertilizer is an effective way to improve the Se nutrition status of crops in Se-deficient areas.

Conducting experimental research on Se enrichment in RR, which can be used as both food and feed, is highly important for several reasons. The first is the study of the physiological and biochemical effects of Se on RR itself, the second is the significance of Se promoting the improvement of grain quality to produce functional foods, and the third is the significance of improving forage quality to promote animal husbandry production. The results of this experiment have positive effects on all three aspects mentioned above. Compared with the numerous studies on inorganic Se, few studies have focused on the influence of foliar spraying on the uptake of organic Se and nano-Se in RR plants. With the development of nanotechnology, safer nanoscale Se has become a focus of attention [50,51]. Nano-Se, as a new form of Se, has advantages such as small size and spherical shape, which are more conducive to crossing cell membranes and entering cells. Moreover, it has advantages such as high safety and good biological activity [51]. One of the purposes of this study was to understand which of the three exogenous Se sources is most suitable for the production of RR.

The results of this research indicate that the foliar spraying of Se can promote the nutritional quality of crop grains, and similar results have also been reported [52,53]. Some researchers even reported a possible increase in yield with the introduction of this element [49,54]. In general, this effect may stem from Se indirectly increasing the chlorophyll content, increasing the leaf photosynthetic rate and stimulating an increase in antioxidant enzyme system activity [55], resulting in an increase in sugar accumulation at the same time [56]. Simultaneously, Se plays a role in the carbohydrate and secondary metabolism of proteins, influencing the activity of enzymes. Consequently, this leads to increased biomass and protein content in RR grains, thereby enhancing crop nutritional quality [57,58]. In this study, the 1000-grain weight initially increased but then decreased with foliar Se application in combination with the T-SS concentration combination. However, these results may be attributed to the excessive Se doses in the body of the plant exerting mild toxic effects and reducing the accumulation of carbohydrates in the grains. This observation suggests that the maximum Se dosage in this experiment may have reached a significantly high level. These findings may indicate that the effect of exogenous Se on plant growth is closely related to the Se type and Se application level [59]. In the present study, compared with the control treatment, the optimal T-SS treatment application increased the biomass and grain yield by 16.57% and 14.55%, respectively (Table 7). The heights of plants enriched with different Se sources increased from 10.96% (900 μg·L^−1^ of NS) to 13.13% (sodium selenite 100 mg·L^−1^) (Table 1), confirming the findings of previous researchers [60,61,62].

The application of Se not only enhances the nutritional value of crops but also influences the absorption of Se and other mineral elements. This result revealed that (as shown in Table 2) the T2-SS treatment resulted in greater Se enrichment than the other two Se treatments did. This difference may be due to the role played by Se in plant growth and the protection of the photosynthetic capacity of plants; thus, Se may be considered an advantageous element for plant resistance to abiotic stresses, resulting in high grain yield [63]. This study revealed that in the ground stems and leaves harvested in the early stage, the Se concentration in the T2-SS combination was 0.929 mg·kg^−1^ and in the grains, the Se content reached 0.757 mg·kg^−1^ in the T2-SS combination. As the treatment concentration increased, the Se content in the grains actually decreased. For both the T-SeMet and T-NS treatments, the highest Se content of the grains occurred in the high-concentration treatment combination but still lower than that in the T-SS. These difference effects may be attributed to variations in Se fertilizers, application doses, and crop tolerances to Se [14,17,64]. Further analysis of the results (Table 3 and Table 4) revealed that the micronutrients in RR were promoted by exogenous selenium. The Ministry of Agriculture of China has clear requirements for the quality indicators of feed in the latest national feed standards, for example, soybean meal requires a crude protein content of over 44%, whereas corn and straw feed require a crude protein content between 7% and 9% because crude protein is an essential nutrient in animal bodies, and different types of feed materials have different requirements for crude protein. Our study revealed that the T3-SS combination increased the crude protein by 6.04% of compared with that of the control, with the starch content increases by 3.69% (Table 3). Lu et al. reported that low Se doses promote plant growth and increases the uptake of trace elements such as Fe, Mn, Cu, and Zn [49]. This experiment also demonstrated this characteristic. Our results (Table 4) indicated that RR grains presented the highest Cu and Fe contents, followed by Mn and Zn. Different doses of SeO_3_^2−^ exert distinct effects on the absorption of cations such as Fe^2+^, Mn^2+^, Cu^2+^, and Zn^2+^ [65,66]. A comparison of the effects of different concentrations of Se from the same exogenous Se source revealed (Table 4) that the contents of trace elements decreased with increasing Se concentration, which was consistent with the findings of studies by Prom-U-Thai et al. (2020) [67] and Cunha et al. (2022) [68] that higher doses may be toxic to rice and reduce the absorption of trace elements. Notably, according to the latest requirements for Se content in Se-enriched agricultural products in southern China (T/HNFX 001—2017) [69], the Se content in rice should be ≤1.0 mg∙kg^−1^. The results of this experiment did not exceed the standard and were Se-enriched and safe.

We showed that the effects of the three Se sources on RR photosynthesis are different, in terms of Pn, the order of effect size was: SS > Met > NS; whereas in the most effective sodium selenite treatment, the effect of each concentration was: T2 >T3 >T1 >T0. This founding is in agreement with the results of Hu et al. [65], who reported that the values of photosynthesis (Pn and Gs) and the parameters of root morphology under selenite and SeMet treatment were greater than those with selenate treatment (P was supplied at 0.1 mmol·L^−1^). Our experiment also revealed an increase in chlorophyll content after Se treatment, which is the basis for increased photosynthesis. Compared with that of the control plants, the SPAD value significantly increased in response to the low-Se treatment with foliar sprays of three types of exogenous Se under the lower concentration of Se, increasing the SPAD value by 19.41%, 24.1%, and 22.01%, respectively (Table 1). These results confirm the conclusions of previous studies [15], and the Se enhances the biosynthesis of photosynthetic products in plants, promoting the repair of chloroplasts damaged by environmental stress and ROS [17]. Studies have indicated that the potential increase in the respiration rate attributed to Se application might contribute to an increase in the biosynthesis of photosynthetic products and increased photosynthetic pigments, stimulating the antioxidant system and reducing ROS formation and resulting in increased RR yields (Table 1 and Table 7). A nano-Se formulation improved the entire physiological apparatus and promoted the development of Chinese cabbage [66]. The application of Se to other crops had effects such as increasing productivity and the generation of a greater number of large tubers in potatoes [70], increasing the functional component contents of waxy maize grains [49], improving the grain yield of canola by approximately 283.5% [71], and increasing the cowpea yield by 1.6 times [72]. Nno-Se 5000 at a foliar concentration of 160 mg·Se L^−1^ resulted in a relatively high coffee yield [73].

### 4.2. Exogenous Application of Se Promoted the Antioxidant Properties of RR

Foliar application of Se at concentrations ranging from 50 to 150 mg·L^−1^ decreased lipid peroxidation and the concentration of hydrogen peroxide, but increased SOD, APX, CAT, and GR activities in RR leaves. These results indicate that foliar Se application stimulates antioxidative metabolism to mitigate reactive oxygen species. Strong evidence indicates that Se can increase the activity of plant antioxidant enzymes under stress conditions [17,44,45]. The antioxidant enzymes SOD, CAT, and GR were selected on the basis of their responses in a number of reports and the role of Se during environmental stress in RR plants. These enzymes exhibited varied responses among Se applications (Figure 1, Figure 2 and Figure 3). As shown in Figure 1A, POD activity was significantly affected by stage, and was significantly affected by treatment concentration. SOD activity (Figure 1B) was greater in the three stages than in the other stages, and the differences in enzyme activity among the multiple treatments in the first and second stages were relatively small, whereas the differences increased in the third stage. The GR activity (Figure 2B) varied slightly between the first and second stages, although there were significant differences among the treatment combinations. Compared with that in the CK treatment, the GR activity in the leaves in the three stages was more pronounced. Nevertheless, in the first and second stages, the GR activity was low, but the GR activity was more than twice as high in the leaves of the three treatment groups than in those of the control group. RR plants treated with T-SS or T-NS in the third stage presented considerably greater GR activity. In terms of nonenzymatic compound content, three Se sources promoted an increase in the GSSG content (Figure 3B). These results suggest that Se has beneficial effects on the increase in photosynthetic pigments and antioxidative metabolism and increased the RR yield and nutritional quality of grains. This finding is highly consistent with the findings of Yu et al. [74] and Gu et al. [75], who demonstrated that SOD, POD, and CAT activities were significantly increased in redwood fruits and aromatic rice after nano-Se treatment. Notably, nanoselenium has a significant effect on plant antioxidant enzyme systems, with multiple enzymes exhibiting strong enzyme activity in stages 2 and 3. These findings seem to indicate that nano-Se has a significant effect on promoting enzyme activity. Because the small size, low toxicity, and high compatibility of nano-Se make the application effect of nano-Se better than that of other forms of Se [76], nano-Se can produce a better spatial distribution of active ingredients on the surface of leaves [77]. Therefore, Hu. et al. reported that rice can quickly convert biological nano-Se into organic Se and store it in plant tissues [35]. This result is consistent with the findings in fragrant rice [55,75].

### 4.3. The Potential of Foliar Biofortification to Enhance the Functional Component Contents of RR Grains

However, in current rice production, improving both yield and quality is an important goal, especially in the production of RR by with harvesting both feed and grain, and quality indicators are an important aspect [78]. The rapid development of agricultural science and technology and improvements in living standards have led to changes in the structure of human diets. To meet these changing demands, Se-enriched functional foods have emerged as a result, and a series of crop varieties with special traits such as waxy maize [49], fragrant rice [55,75], and wheat cultivars (colored-grain wheat) [79], have so been developed.

There are many examples of crop quality improvement and yield increase resulting from the application of exogenous Se. These include waxy maize [49], naked oats [54], fragrant rice [55,75], wheat [65], sorghum [59], soybeans [80], and various vegetables [81,82]. There must be important nutrients in these high-quality products that are most beneficial to the human body. In most grains, the main Se species is SeMet [80]. Here, our founding that SeMet was the main Se species produced in the brown rice corroborate the results of Deng et al. (2021) [25] (Figure 4). Notably, when the Se source was organic Se, the content of organic Se in the rice grains and leaves was high, and the type of protein Se changed. SeMeCys was present in only the organic Se treatment (T-SeMet), and the content of SeMeCys in the leaves and grains was 3.52% and 2.39%, respectively. Kowalska et al. (2023) [82] reported that SeMet was more effective at biofortifying than Na_2_SeO_3_ was, while those treated with Na_2_SeO_3_ or Na_2_SeO_3_ + SA also accumulated MeSeCys and selenite (SeO_3_^−2^) which is consistent with the findings of Yuan et al. (2023) [15]. This experiment yielded similar results, with RR grains appearing as SeMeCys at T2-SeMet and T3-SeMet and appearing simultaneously in the leaves and grains. Figure 4 shows that organic Se with T2-SeMet presented significantly higher MeSet levels than the other two types of exogenous Se (Table S1). This result indicates that more human-usable organic Se exists in organic Se treatment than in other Se species and is an important factor in determining the value of Se biofortification products [15,81,82]. Notably, different Se sources and concentrations have different treatment effects on different plants, and the reasons for these effects are multifaceted. However, there are three main aspects: first, different plant characteristics [40,81,83]; second, different application methods and environmental impacts [46,47]; and third, different in measurement methods [84].

On the basis of the above discussion, from the perspective of producing functional foods, organic Se is the best selenium source for Se biofortification [80]. However, from the perspective of production cost, selenite is the most economical, and it can also produce SeMeCys in other crops [82]. Other factors include the involvement of various doses associated with application stages and concentrations and the variety type of the crops themselves. The interactions between Se and diverse plant species of crops remain controversial and require further study [14,15,17]. As a resource-efficient and green method of producing more rice grains, it has been widely adopted in several rice-growing countries such as the United States, Japan, Indonesia, and India [85]. In the future, more research should be conducted at the physiological and molecular levels of plant absorption to reveal the regulatory mechanisms by which different Se sources affect crop yield formation and improve grain quality.

In rice, starch is the main stored carbohydrate that affects its quality. RR can increase the amylose content, phenolic lipid, and lysine contents, but decreases the protein content and gel consistency [86]. This founding is undoubtedly a significant benefit for researchers in the development of functional foods. The concept of resistant starch (RS) was proposed in the 1980s by the British physiologist Englyst [87]. The International Rice Research Institute analyzed the starch structure of different rice varieties and reported that RS is highly related to amylose [88]. High RS rice lines contain not only a high content of amylose but also a high proportion of long-chain branched amylopectin, which is prone to forming chain-to-chain hydrogen bonds, resulting in a high content of RS and the high thermal stability of starch granules. This is the intrinsic reason why rice materials with little difference in amylose content have different RS contents. These findings provide new breeding indicators for functional rice varieties with high RS contents [89].

Research has confirmed that foods rich in RS have the effects of lowering the glycemic index, increasing satiety, preventing blood sugar-related diseases, and preventing intestinal-related diseases [90]. Yadav et al. (2010) [91] noted that the RS content in conventionally cultivated rice is usually <1%, which is far below the daily recommended RS intake for humans. Our study revealed an increase in the amylose starch content (AC) following Se foliar spraying (Table 11). The AC value of the brown rice treatment combination with the greatest increase was that of T2-SS, with an increase of 62.97%. The reason directly related to the increase in AC is that the RS value has also significantly increased. The combination with the maximum increase in the RS value was that the T2-SS increased by 435.90%. The results of this research indicate that exogenous Se has very positive effects not only on grain yield but also on grain quality. This finding is in line with the results of other studies involving various crops and Se doses (up to 120 g·ha^−1^) [1,2,3,12,77]. Our research is consistent with the findings of Yuan et al. [15], who reported that the additional application of Se increased the amylose content by 6.18% over that of the CK. Therefore, breeding rice rich in RS is an important route for improving rice varieties, as reported in a previous study [92]. Another important route should be foliar Se application, which is time-saving and labor-saving and has significant effects. This genetic improvement provides a new strategy for breeding novel rice varieties with high RS contents.

### 4.4. Biostatistical Analysis

By using correlation analysis techniques, we can obtain a clearer understanding of the relationships between various traits and scientifically evaluate the various properties of materials. Therefore, we performed a correlation analysis of 11 nutritional quality indicators of RR under various Se doses. Our analysis revealed significant correlations among nutritional components, trace elements, and grain quality traits.

The Se levels of RR grains were significantly positively correlated with organic Se, protein Se, Fe, and amylose contents and other indicators (Figure 5). These observations indicate that Se accumulation may promote the accumulation of trace elements and functional components, which aligns with the findings of Yuan et al. (2023) [15]. Principal component analysis serves as an objective, simple, and rapid evaluation system that effectively reflects the comprehensive quality of RR grain following Se foliar spraying [93]. Similarly, the results of Lu et al. (2024) [49] are confirmed our findings. This study revealed that T-SS presented relatively high levels of protein Se, resistant starch, amylose content, amino acid, and other nutrients, whereas T-MeSe presented relatively high contents of organic Se, especially high levels of functional components (SeMeCys) (Figure 4). The beneficial Se source treatment led to more abundant nutrients, whereas the disadvantageous Se source treatment yielded relatively few nutrients.

## 5. Conclusions

Our study demonstrated that the foliar spraying of Se increased the levels of nutritional substances, trace elements, and functional components in RR grains and straw during the tillering stage. In conclusion, the distribution and accumulation of Se in RR plants are different for different concentrations of exogenous Se and different Se spray concentrations. The application of Se effectively promoted the nutrient quality of RR, including total Se, protein Se, trace element, and RS contents, as well as the activity of antioxidant enzymes (SOD, POD, CAT, and GR) and the content of nonenzymatic proteins (GSH-Px and GSSG) in brown rice. In the present study, Se increased the total Se content in leaves and grains, which was enhanced by the plant photosynthesis, resulting in increased biomass and yield as the main products of photosynthesis. All the treatments showed that the grain reaches a Se concentration in the grain reached more than 200 µg·kg^−1^. Moreover, the Se content of rice straw is also relatively high. Irrespective of the total Se, protein Se, and organic Se accumulation were greater in the sodium selenite and SeMet treatments. Compared with the nano-Se treatment, the three tested exogenous Se treatments caused no harm to the plant development, had no impact on grain yield but increased biomass, increased grain quality, increased RS content, and increased SeMeCys concentration, which is a representative component. PCA revealed that the nutritional quality of the selenite treatments surpassed that of the other treatments. Both grains and straw exhibited optimal quality characteristics under the selenite treatment, remaining within the safe range for Se-enriched agricultural products. The findings of this study may provide valuable insights for the implementation of RR in other rice-producing regions worldwide to improve productivity and environmental performance in current rice production.

## Figures and Tables

**Table 1:** The effects of three exogenous Se treatments on the growth and grain traits of RR.

Treatment	Seedling Height (cm)	Chlorophyll Value	Adult Plant Height (cm)	Spike Length (cm)	Number of Grains per Spike	1000-Grain Weight (g)
CK	80.07 ± 1.25 g	38.17 ± 2.16 e	114.82 ± 9.2 e	23.75 ± 6.11 c	20.58 ± 3.54 d	17.40 ± 3.01 c
T1-SS	83.97 ± 2.72 e	44.58 ± 2.6 b	120.37 ± 7.3 c	25.24 ± 5.61 b	25.58 ± 3.5 ab	16.60 ± 1.17 db
T2-SS	93.40 ± 3.62 b	45.27 ± 4.11 a	126.62 ± 7.23 a	24.92 ± 3.68 b	26.05 ± 4.33 a	20.11 ± 3.17 b
T3-SS	83.95 ± 3.21 e	44.37 ± 3.25 c	116.73 ± 6.25 d	23.51 ± 6.1 c	26.28 ± 4.36 a	17.41 ± 1.56 c
T1-SeMet	90.40 ± 2.18 c	44.15 ± 4.2 c	110.05 ± 8.8 f	24.55 ± 7.62 b	24.60 ± 5.12 b	18.43 ± 2.2 c
T2-SeMet	84.15 ± 3.11 e	47.37 ± 3.52 a	126.12 ± 7.23 a	22.06 ± 5.02 d	26.64 ± 6.13 a	17.78 ± 3.27 c
T3-SeMet	86.70 ± 3.32 d	45.15 ± 5.3 ab	122.71 ± 4.34 b	27.46 ± 8.21 a	24.12 ± 3.43 c	16.23 ± 2.19 d
T1-NS	94.75 ± 5.27 a	42.76 ± 3.41 d	122.80 ± 6.27 b	27.37 ± 6.2 a	23.29 ± 7.2 c	22.31 ± 3.26 a
T2-NS	82.21 ± 2.34 f	45.83 ± 7.03 a	116.90 ± 3.5 d	24.26 ± 4.4 c	23.48 ± 4.33 c	20.00 ± 2.15 b
T3-NS	95.60 ± 5.17 a	44.39 ± 6.41 bc	116.55 ± 11.2 d	24.38 ± 6.33 b	24.51 ± 7.14 b	15.81 ± 1.97 de

Note: RR—ratooning rice. The treatments were set as follows: T0: the control treatment (CK, distilled water only); T1-SS at a low rate (50 mg·L^−1^ of SS), T2-SS at a medium rate (100 mg·L^−1^ of SS), and T3-SS at a high rate (150 mg·L^−1^ of SS); T1-SeMet at a low rate (50 mg·L^−1^ of SeMet), T2-SeMet at a medium rate (100 mg·L^−1^ of SeMet), and T3-SeMet at a high rate (150 mg·L^−1^ of SeMet); and T1-NS at a low rate (150 μg·L^−1^ of NS), T2-NS at a medium rate (450 μg·L^−1^ of NS), and T3-NS at a high rate (900·μg L^−1^ of NS). The lowercase letters in the table represent significant differences at the 0.05 level, the same letters represent non-significant differences, and different letters represent significant differences; the data are presented as the means ± SDs (n = 3). The same applies below.

**Table 2:** Se contents in different parts of LY plants sprayed with exogenous Se on leaves during tillering stage.

Treatments	Roots (mg·kg^−1^)	Straw (mg·kg^−1)^	Spike Axis (mg·kg^−1^)	Grains (mg·kg^−1^)
CK	0.276 ± 0.02 f	0.078 ± 0.01 i	0.068 ± 0.01 f	0.063 ± 0.02 f
T1-SS	0.431 ± 0.23 c	0.356 ± 0.12 g	0.236 ± 0.05 d	0.295 ± 0.1 d
T2-SS	0.657 ± 0.52 b	0.929 ± 0.33 a	1.048 ± 0.42 a	0.757 ± 0.2 a
T3-SS	0.368 ± 0.33 d	0.516 ± 0.21 e	0.356 ± 0.13 c	0.542 ± 0.12 b
T1-SeMet	0.405 ± 0.42 cd	0.247 ± 0.08 h	0.117 ± 0.03 e	0.137 ± 0.03 e
T2-SeMet	0.468 ± 0.34 c	0.461 ± 0.22 f	0.179 ± 0.03 de	0.402 ± 0.11 c
T3-SeMet	0.332 ± 0.12 d	0.594 ± 0.14 d	0.403 ± 0.13 c	0.263 ± 0.07 d
T1-NS	0.351 ± 0.09 d	0.288 ± 0.05 h	0.126 ± 0.03 e	0.112 ± 0.02 e
T2-NS	0.708 ± 0.22 a	0.684 ± 0.17 c	0.368 ± 0.06 c	0.382 ± 0.06 c
T3-NS	0.327 ± 0.13 e	0.887 ± 0.24 b	0.770 ± 0.15 b	0.256 ± 0.07 d

Note: Different letters indicate a significant difference in the same column at *p* < 0.05. The values are the means ± SDs (n = 3).

**Table 3:** Effects of foliar Se application on the quality of straw feed in the current season of RR.

Treatments	Dry Matter Content (%)	Crude Protein Content (%)	Crude Ash Content (%)	Crude Fiber Content (%)	Starch Content (%)
CK	70.32 ± 6.8 h	8.11 ± 0.21 b	4.23 ± 0.33 c	8.40 ± 0.85 c	65.84 ± 3.21 c
T1-SS	69.39 ± 5.42 g	7.86 ± 0.7 b	3.66 ± 0.74 d	8.86 ± 0.77 b c	62.31 ± 4.82 d
T2-SS	89.22 ± 5.43 a	7.96 ± 0.41 b	5.01 ± 0.28 b	9.62 ± 0.8 a	68.27 ± 6.11 a
T3-SS	88.15 ± 3.41 b	8.60 ± 0.21 a	4.12 ± 0.34 c	9.06 ± 1.32 b	67.60 ± 5.31 a
T1-SeMet	71.38 ± 6.42 g	7.90 ± 0.42 b	5.20 ± 0.43 b	6.34 ± 0.65 e	66.86 ± 4.32 b
T2-SeMet	77.67 ± 5.76 e	7.93 ± 0.23 b	3.91 ± 1.1 c d	5.71 ± 0.63 f	65.24 ± 3.51 c
T3-SeMet	73.84 ± 7.32 f	5.97 ± 0.2 d	5.02 ± 0.2 b	8.44 ± 0.75 c	63.21 ± 5.06 d
T1-NS	79.80 ± 6.05 d	6.75 ± 1.1 c	4.31 ± 0.73 c	7.97 ± 1.01 d	61.78 ± 6.7 d
T2-NS	81.48 ± 7.52 c	5.33 ± 0.57 e	4.61 ± 0.27 c	8.02 ± 0.21 d	61.79 ± 6.44 d
T3-NS	77.51 ± 8.45 e	5.87 ± 0.66 d	5.60 ± 0.7 a	8.30 ± 2.3 d	66.03 ± 3.33 b

Note: Different letters indicate a significant difference in the same column at *p* < 0.05. The values are the means ± SDs (n = 3).

**Table 4:** Effects of foliar Se application on the contents of microelements in of straw feed during the current season in the RR.

Treatments	Iron (mg·kg^−1^)	Copper (mg·kg^−1^)	Zinc (mg·kg^−1^)	Manganese(mg·kg^−1^)
CK	29.35 ± 3.25 h	38.06 ± 4.31 c	6.84 ± 0.7 e	22.2 ± 1.21 d
T1-SS	46.38 ± 2.17 b	41.07 ± 1.74 b	8.53 ± 2.27 c	31.53 ± 2.01 a
T2-SS	49.16 ± 3.26 a	39.53 ± 2.35 b	10.33 ± 1.21 b	18.6 ± 3.01 e
T3-SS	37.32 ± 6.13 e	38.61 ± 7.21 c	11.3 ± 2.07 a	29.33 ± 3.61 b
T1-SeMet	41.66 ± 4.23 c	38.63 ± 3.65 c	7.36 ± 1.02 d	26.8 ± 4.2 c
T2-SeMet	37.86 ± 8.01 e	35.43 ± 5.24 e	8.12 ± 0.67 c d	23 ± 3.4 d
T3-SeMet	33.85 ± 3.56 f	35.59 ± 3.83 e	7.86 ± 1.2 d	21.55 ± 3.3 d
T1-NS	30.82 ± 6.13 g	42.4 ± 6.1 a	11.06 ± 3.02 a	18.32 ± 2.41 e
T2-NS	41.48 ± 4.02 c	31.55 ± 4.26 f	8.33 ± 2.05 c	18.9 ± 2.3 e
T3-NS	39.85 ± 2.42 d	37.33 ± 33.6 d	7.98 ± 1.15 d	26.1 ± 4.38 c

Note: Different letters indicate a significant difference in the same column at *p* < 0.05. The values are the means ± SDs (n = 3).

**Table 5:** Effects of foliar Se application on the photosynthesis parameters during the current season in RR.

Treatments	Pn (μmol·CO_2_·m^−2^·s^−1^)	Ci (μmol·mol ^−1^)	Tr (mmol H_2_O·m^−2^·s^−1^)	Gs (μmol·m^−2^·s^−1^)
CK	19.31 ± 0.05 c	110.21 ± 0.06 d	3.47 ± 0.20 c	0.35 ± 0.02 b
T1-SS	20.93 ± 0.05 c	52.46 ± 0.72 f	5.08 ± 0.1 a	0.36 ± 0.01 b
T2-SS	23.25 ± 0.04 a	25.79 ± 1.2 h	2.33 ± 0.01 d	0.48 ± 0.01 a
T3-SS	21.84 ± 0.03 b	52.78 ± 0.46 f	2.48 ± 0.01 d	0.29 ± 0.00 c
T1-SeMet	21.83 ± 0.03 b	121.75 ± 0.13 b	4.63 ± 0.02 ab	0.36 ± 0.00 b
T2-SeMet	17.89 ± 0.06 d	107.39 ± 1.21 e	4.84 ± 0.7 a	0.25 ± 0.00 d
T3-SeMet	10.22 ± 0.04 f	106.57 ± 0.16 e	1.84 ± 0.02 e	0.21 ± 0.00 e
T1-NS	19.36 ± 0.05 c	42.68 ± 0.17 g	1.79 ± 0.02 e	0.18 ± 0.01 f
T2-NS	17.09 ± 0.02 e	125.66 ± 0.21 a	1.13 ± 0.03 f	0.18 ± 0.02 f
T3-NS	19.32 ± 0.14 c	117.66 ± 0.14 c	3.96 ± 0.02 b	0.12 ± 0.01 g

Note: photosynthetic rate (Pn), intercellular CO_2_ concentration (Ci), transpiration rate (Tr), stomatal conductance (Gs). The bars represent the standard deviations (SDs) of the means (n = 3). Different letters indicate significant differences among treatments at *p* < 0.05.

**Table 6:** Effects of foliar Se application on the photosynthesis in the subsequent RR season.

Treatments	Pn (μmol·CO_2_·m^−2^·s^−1^)	Ci (μmol·mol^−1^)	Tr (mmol·H_2_O·m^−2^·s^−1^)	Gs (μmol·m^−2^·s^−1^)
CK	17.02 ± 0.04 d	113.41 ± 0.08 c	4.54 ± 0.06 a	0.34 ± 0.02 b
T1-SS	21.52 ± 0.03 a	51.88 ± 0.14 i	4.38 ± 0.02 ab	0.34 ± 0.01 b
T2-SS	20.23 ± 0.12 b	81.77 ± 0.64 f	2.13 ± 0.02 e	0.11 ± 0.01 e
T3-SS	17.12 ± 0.03d	104.55 ± 0.17 e	1.80 ± 0.02 f	0.31 ± 0.00 c
T1-SeMet	18.73 ± 0.03 c	63.38 ± 0.71 g	4.36 ± 0.01 b	0.38 ± 0.00 b
T2-SeMet	16.28 ± 0.08 e	105.64 ± 1.15 e	3.44 ± 0.21 c	0.37 ± 0.00 b
T3-SeMet	21.44 ± 0.05 a	55.97 ± 1.14 h	1.95 ± 0.02 e	0.21 ± 0.00 d
T1-NS	18.66 ± 0.03c	111.54 ± 0.12 d	2.49 ± 0.01 d	0.19 ± 0.01 d
T2-NS	19.86 ± 0.05 b	117.35 ± 0.14 b	2.21 ± 0.01 e	0.18 ± 0.01 d
T3-NS	19.12 ± 0.02 c	127.83 ± 0.27 a	4.74 ± 0.0 a	0.44 ± 0.01 a

Note: photosynthetic rate (Pn), intercellular CO_2_ concentration (Ci), transpiration rate (Tr), stomatal conductance (Gs). The bars represent the standard deviations (SDs) of the means (n = 3). Different letters indicate significant differences among treatments at *p* < 0.05.

**Table 7:** Grain yield and total biomass of LY rice with exogenous Se foliar spraying.

Treatments	Total Biomass (t·ha^−1^)	Grain Yield (t·ha^−1^)
Se source (S)		
CK	17.21	6.94
T1-SS	17.13	7.69
T2-SS	18.42	8.53
T3-SS	17.16	7.63
T1-SeMet	17.76	6.68
T2-SeMet	17.95	5.83
T3-SeMet	15.43	7.21
T1-NS	16.56	7.08
T2-NS	16.62	6.98
T3-NS	16.97	7.11
LSD (*p* = 0.05)	1.36	**NS**
Concentration (C)		
T1-SS	17.13	7.69
T1-SeMet	17.76	6.68
T1-NS	16.56	7.08
T2-SS	18.42	8.53
T2-SeMet	17.95	5.83
T2-NS	16.62	7.11
T3-SS	17.16	7.63
T3-SeMet	15.43	7.21
T3-NS	16.97	7.11
LSD (*p* = 0.05)	**NS**	**NS**
S × C	*	**NS**

Note: NS indicates non-significance; * significant at *p* < 0.05.

**Table 8:** Organic Se and protein Se concentrations and proportions at the tillering stage with exogenous Se foliar spraying.

Treatments	Organic Se (mg·kg^−1^)	Proportion(%) Organic Se/Total Se	Protein Se (mg·kg^−1^)	Proportion(%) Protein Se/Total Se
Se Source(S)–Concentration(C)				
CK	0.055	79.84	0.0264	44.18
T1-SS	0.281	90.00	0.1328	42.54
T2-SS	0.817	90.62	0.328	36.40
T3-SS	0.575	88.75	0.3064	40.68
T1-SeMet	0.152	89.25	0.061	43.24
T2-SeMet	0.256	85.22	0.1425	46.32
T3-SeMet	0.363	90.52	0.1383	38.61
T1-NS	0.144	87.23	0.0684	44.22
T2-NS	0.239	85.36	0.1232	41.14
T3-NS	0.367	90.28	0.1466	33.86
LSD (*p* = 0.05)	0.168	5.14	0.0795	5.89
S × C	NS	NS	NS	NS

Note: NS indicates non-significance.

**Table 9:** Effects of foliar spraying of exogenous Se on trace element contents in RR brown rice.

Treatments	Zinc (mg·kg^−1^)	Iron (mg·kg^−1^)	Copper (mg·kg^−1^)	Manganese (mg·kg^−1^)
CK	14.22 ± 0.31 e	16.41 ± 0.52 e	9.36 ± 1.85 a	16.97 ± 3.42 b
T1-SS	17.18 ± 0.6 b	17.44 ± 1.06 d	6.48 ± 0.57 d	16.43 ± 1.22 b
T2-SS	15.62 ± 0.36 d	21.77 ± 3.22 a	8.33 ± 0.1 b	18.87 ± 0.52 a
T3-SS	15.91 ± 0.12 c	21.05 ± 1.6 a	8.33 ± 0.01 b	18.56 ± 0.16 a
T1-SeMet	15.13 ± 0.53 d	19.30 ± 0.84 c	7.66 ± 0.0.12 c	10.45 ± 0.81 e
T2-SeMet	14.11 ± 0.23 e	18.93 ± 3.43 c	9.68 ± 0.52 a	13.93 ± 1.64 cd
T3-SeMet	13.52 ± 0.12 f	20.55 ± 2.03 b	8.89 ± 0.33 b	12.75 ± 1.67 d
T1-NS	16.18 ± 1.54 c	19.27 ± 1.61 c	8.12 ± 0.64 bc	14.62 ± 0.47 c
T2-NS	18.65 ± 0.94 a	21.88 ± 0.41 a	7.39 ± 0.36 c	14.36 ± 0.71 c
T3-NS	17.38 ± 0.67 b	20.02 ± 3.21 b	8.35 ± 0.3 b	12.56 ± 1.24 d

Note: Different letters indicate a significant difference in the same column at *p* < 0.05. The values are the mean ± SDs (n = 3).

**Table 10:** Effects of foliar Se application on the processing and appearance of RR brown rice.

Treatments	Processing Quality			Appearance Quality		
	Brown Rice Rate/%	Milled Rice Rate/%	Head Milled Rice Rate/%	Chalkiness Rate/%	Chalkiness Size/%	Chalkiness Degree/%
CK	83.21 a	76.20 c	65.91 c	27.02 a	19.61 a	3.22 c
T1-SS	82.48 b	75.01 c	46.90 g	18.20 d	10.33 h	3.06 c
T2-SS	81.82 c	79.37 a	68.41 a	16.54 f	12.05 g	2.04 d
T3-SS	82.34 b	74.91 c	54.13 e	17.06 e	13.03 f	1.63 f
T1-SeMet	83.02 a	75.53 c	52.05 e	25.47 b	15.50 d	3.91 a
T2-SeMet	82.83 a	75.12 c	51.14 f	19.05 c	13.32 f	2.02 d
T3-SeMet	81.64 c	73.93 d	52.81 f	17.51 e	16.73 c	1.87 e
T1-NS	82.16 b	74.51 d	55.30 e	20.04 b	17.51 b	3.54 b
T2-NS	83.22 a	77.03 b	66.80 b	12.06 h	16.75 c	2.03 d
T3-NS	82.14 b	75.92 c	59.41 d	17.62 e	14.54 e	2.14 d

Note: Different letters indicate significant differences (*p* < 0.05). The data are represented as means ± SEs (n = 3).

**Table 11:** Effects of foliar application of different amounts of exogenous Se on the cooking and eating quality of RR brown rice.

Treatments	RS (%)	AC (%)	GC (mm)	GT (ASV)	AA (µg·g^−1^)	Antioxidant Activity (% Inhibition)
CK	0.78 ± 0.12 h	14.8 ± 1.84 g	50.00 ± 3.86 g	1.93 ± 0.33 bc	8.95 ± 1.02 b	38.66 ± 1.73 d
T1-SS	0.86 ± 0.31 g	19.18 ± 3.6 d	66.45 ± 3.62 f	1.78 ± 1.46 d	7.84 ± 2.33 e	46.58 ± 3.47 a
T2-SS	2.76 ± 0.32 c	24.12 ± 5.23 a	75.55 ± 4.5 d	2.71 ± 2.31 a	8.55 ± 0.64 c	28.76 ± 5.13 g
T3-SS	1.86 ± 0.12 d	22.91 ± 3.21 b	77.43 ± 6.14 c	2.15 ± 1.6 b	7.83 ± 2.02 e	28.46 ± 3.43 g
T1-SeMet	3.58 ± 0.62 b	16.83 ± 2.76 f	79.24 ± 6.41 b	1.39 ± 0.14 e	9.74 ± 0.68 a	44.54 ± 2.77 b
T2-SeMet	4.18 ± 0.73 a	20.60 ± 4.63 d	67.36 ± 6.3 f	1.83 ± 0.75 c	6.78 ± 1.32 f	33.35 ± 4.34 f
T3-SeMet	1.27 ± 0.33 e	21.44 ± 5.2 c	78.24 ± 6.14 b	2.06 ± 0.42 b	8.55 ± 2.07 c	32.57 ± 4.7 f
T1-NS	0.98 ± 0.32 f	18.71 ± 5.71 e	80.37 ± 1.53 a	1.29 ± 0.17 e	8.22 ± 1.46 d	34.66 ± 5.74 e
T2-NS	0.85 ± 0.11 g	17.88 ± 4.2 e	71.76 ± 6.16 e	2.81 ± 0.57 a	9.73 ± 1.72 a	24.57 ± 1.22 h
T3-NS	0.71 ± 0.09 g	23.13 ± 4.35 c	83.24 ± 5.25 f	2.06 ± 0.23 b	8.58 ± 0.41 c	42.64 ± 3.76 c

Note: RS—resistant starch; AC—amylose content; GC—gelatinization consistency; GT—gelatinization temperature; ASV—alkali spreading value; AA—amino acid. Different letters indicate significant differences (*p* < 0.05). The data are represented as means ± SEs (n = 3).

## Data Availability

All data generated or analyzed during this study are included in this published article.

## Supplementary Materials

The following supporting information can be downloaded at: https://www.mdpi.com/article/10.3390/foods13223637/s1, Table S1. The content of Se speciation forms and total Se in rice leaves and grains (mg·(kg·D.W.)^−1^); Table S2. The correlation matrix eigenvalues and corresponding matrix eigenvectors of nutritional quality, trace elements, and pigment content traits of RR; Table S3. The principal component scores of nutritional quality, trace element content of RR grains; Figure S1: The Se content in leaves and grains spraying exogenous Se of LY in tillering stage.

## Abbreviations

Se	Selenium
SS	Sodium selenite
SeMet	Selenomethionine
NS	Nanoselenium
CK	Control
T0	The control treatment (CK, only distilled water)
T1-SS	50 mg L^−1^ of SS
T2-SS	100 mg L^−1^ of SS
T3-SS	150 mg L^−1^ of SS
T1-SeMet	50 mg L^−1^ of SeMet
T2-SeMet	100 mg L^−1^ of SeMet
T3-SeMet	150 mg L^−1^ of SeMet
T1-NS	150 μg L^−1^ of NS
T2-NS	450 μg L^−1^ of NS
T3-NS	900 μg L^−1^ of NS
CAT	Catalase
SOD	Superoxide dismutase
POD	Peroxidase
GR	Glutathione reductase
GSSG	Oxidized glutathione
APX	Ascorbate peroxidase
GSH	Reduced glutathione
Pn	Photosynthetic rate
Ci	Intercellular CO_2_ concentration
Tr	Transpiration rate
Gs	Stomatal conductance
AC	Amylose content
GC	Gelatinization consistency
ASV	Alkali spreading value
GT	Gelatinization temperature
AA	Amino acid
RR	Ratooning rice
RS	Resistant starch
MeSeCys	Methylselenocysteine
PCA	Principal component analysis
LY	Liangyou 6326
