# Supplementary material for: Enhancement of Nutrient, Trace Element, and Organic Selenium Contents of Ratooning Rice Grains and Straw Through Foliar Application of Selenite
Source: Foods. 2024 Nov 14;13(22):3637. doi: 10.3390/foods13223637 (PMC11594030; doi:10.3390/foods13223637)
Supplement: Supplementary file 1 [file foods-13-03637-s001.zip › 10-27-Table S1.docx]

Table S1. The content of Se speciation forms and total Se in rice leaves and grains ( mg🞗(kg D.W.)^-1^ )

| Treatment | Total Se content | | | SeMet | | | SeCys2 | | |  |
| --- | --- | --- | --- | --- | --- | --- | --- | --- | --- | --- |
|  | | leaves | grain | | leaves | grain | | leaves | grain | |
| CK | | 0.078±0.041 e | 0.063±0.016 f | | 0.0613±0.012 g | 0.046±0.003 f | | 0.012±0.007 d | 0.098±0.021 b | |
| T1-SS | | 0.356±0.053 c | 0.259±0.113 d | | 0.290±0.018 e | 0.251±0.036 d | | 0.056±0.029 b | 0.036±0.006 c | |
| T2-SS | | 0.929±0.125 a | 0.757±0.131 a | | 0.763±0.044 a | 0.660±0.044 b | | 0.151±0.061 a | 0.089±0.024 b | |
| T3-SS | | 0.506±0.112 b | 0.342±0.067 c | | 0.402±0.035 d | 0.411±0.083 c | | 0.069±0.041 a | 0.099±0.011 b | |
| T1-SeMet | | 0.247±0.033 d | 0.137±0.016 e | | 0.194±0.037 f | 0.121±0.015 e | | 0.031±0.012 c | 0.014±0.001 e | |
| T2-SeMet | | 0.461±0.053 b | 0.402±0.081 b | | 0.397±0.066 d | 0.372±0.085 c | | 0.029±0.006 c | 0.011±0.001 e | |
| T3-SeMet | | 0.594±0.094 b | 0.262±0.068 d | | 0.495±0.065 c | 0.229±0.071 d | | 0.082±0.011 b | 0.024±0.011 d | |
| T1-NS | | 0.288±0.031 d | 0.112±0.011 e | | 0.206±0.053 f | 0.804±0.061 a | | 0.042±0.012 bc | 0.009±0.000 e | |
| T2-NS | | 0.684±0.101 b | 0.382±0.023 c | | 0.503±0.056 c | 0.285±0.032 d | | 0.105±0.031 b | 0.578±0.045 a | |
| T3-NS | | 0.887±0.153 a | 0.256±0.042 d | | 0.624±0.121 b | 0.177±0.022 e | | 0.176±0.013 a | 0.471±0.031 a | |

Note: Means followed by different letters indicate significant differences (p < 0.05). Control—without application of Se.

(Continued table)

| Treatment | SeMeCys | | Se (IV) | | Se(VI) | |
| --- | --- | --- | --- | --- | --- | --- |
|  | leaves | grain | leaves | grain | leaves | grain |
| CK | 0.000±0.000 b | 0.000±0.000 c | 0.047±0.011 a | 0.070±0.002 e | 0.000±0.000 d | 0.000±0.000 b |
| T1-SS | 0.000±0.000 b | 0.000±0.000 c | 0.001±0.000 d | 0.085±0.004 d | 0.000±0.000 | 0.000±0.000 b |
| T2-SS | 0.000±0.000 b | 0.000±0.000 c | 0.015±0.003 c | 0.079±0.014 d | 0.000±0.000 d | 0.000±0.000 b |
| T3-SS | 0.000±0.000 b | 0.000±0.000 c | 0.045±0.005 a | 0.324±0.031 a | 0.000±0.000 d | 0.000±0.000 b |
| T1-SeMet | 0.000±0.000 b | 0.000±0.000 c | 0.022±0.007 b | 0.019±0.011 g | 0.000±0.000 d | 0.000±0.000 b |
| T2-SeMet | 0.016±0.001 a | 0.009±0.000 a | 0.019±0.003 b | 0.102±0.014 c | 0.000±0.000 d | 0.000±0.000 b |
| T3-SeMet | 0.014±0.001 a | 0.004±0.000 b | 0.028±0.011 b | 0.054±0.008 f | 0.000±0.000 d | 0.000±0.000 b |
| T1-NS | 0.000±0.000 b | 0.000±0.000 c | 0.016±0.007 c | 0.052±0.004 f | 0.023±0.001 c | 0.014±0.005 a |
| T2-NS | 0.000±0.000 b | 0.000±0.000 c | 0.044±0.012 a | 0.291±0.033 a | 0.033±0.004 b | 0.011±0.003 a |
| T3-NS | 0.000±0.000 b | 0.000±0.000 c | 0.043±0.011 a | 0.201±0.016 b | 0.043±0.008 a | 0.012±0.002 a |
