# Supplementary material for: Enhancement of Nutrient, Trace Element, and Organic Selenium Contents of Ratooning Rice Grains and Straw Through Foliar Application of Selenite
Source: Foods. 2024 Nov 14;13(22):3637. doi: 10.3390/foods13223637 (PMC11594030; doi:10.3390/foods13223637)
Supplement: Supplementary file 1 [file foods-13-03637-s001.zip › 10-27-Table S2~S3.docx]

Table S2. The correlation matrix eigenvalues and corresponding matrix eigenvectors of nutritional quality, trace elements and pigment content traits of RR

| **Primary Component** | **Principal Component Number** | | | | | |
| --- | --- | --- | --- | --- | --- | --- |
|  | 1 | 2 | 3 | 4 | 5 | 6 |
| Eigenvalue | 2.279 | 1.417 | 1.188 | 1.033 | 0.752 | 0.675 |
| Percentage of variance (%) | 47.203% | 18.252% | 12.830% | 9.696% | 5.140% | 4.137% |
| Cumulative (%) | 47.203% | 65.455% | 78.285% | 87.981% | 93.121% | 97.258% |
| Se | 3.216 | -0.254 | 0.193 | -0.021 | 0.488 | 0.265 |
| Organic Se (O-Se) | 3.159 | -0.176 | 0.198 | -0.217 | 0.491 | -0.780 |
| Protein Se (P-Se) | 3.227 | -0.282 | 0.302 | -0.305 | 0.334 | -0.290 |
| Zn | 1.128 | 2.636 | 0.530 | -0.575 | -0.710 | 0.786 |
| Cu | -0.841 | -2.651 | 0.186 | 1.107 | -0.844 | -0.535 |
| Mn | 0.776 | -1.184 | 2.800 | -0.561 | 0.802 | 0.329 |
| Fe | 2.817 | 1.032 | -0.605 | 1.166 | -0.420 | -0.134 |
| Resistant starch (RS) | 1.288 | -1.260 | -2.324 | 0.243 | 1.066 | 0.971 |
| Amylose content (AC) | 2.980 | -0.070 | -0.579 | -0.621 | -0.751 | -0.892 |
| Amino acid (AA) | -1.164 | 1.923 | 0.339 | 1.873 | 1.221 | -0.820 |
| Antioxidant activity (AnC) | -1.994 | 0.422 | -0.994 | -2.100 | 0.633 | -0.896 |

Table S3. The principal component scores of nutritional quality, trace elements content of RR grains

| Eigenvalue | PC1 | PC2 | PC3 | PC4 | PC5 | PC6 |
| --- | --- | --- | --- | --- | --- | --- |
| CK1 | -2.661 | -0.854 | 1.042 | 0.179 | 0.210 | 0.327 |
| CK2 | -2.691 | -0.957 | 1.045 | 0.223 | -0.190 | -0.017 |
| CK3 | -2.773 | -0.690 | 0.878 | 0.296 | 0.663 | 0.033 |
| T1-SS | -0.196 | 1.330 | 0.370 | -2.630 | 0.392 | -0.372 |
| T2-SS | 4.442 | -0.666 | 0.654 | 0.274 | 1.095 | 0.393 |
| T3-SS | 3.250 | -0.666 | 0.947 | -0.198 | -0.005 | -0.112 |
| T1-MeSe | -1.435 | 1.166 | -2.558 | 0.469 | 1.297 | 0.035 |
| T2-MeSe | 0.872 | -2.591 | -1.765 | -0.270 | -0.600 | -1.079 |
| T3-MeSe | 0.456 | -0.549 | -0.744 | 0.878 | -0.612 | 1.059 |
| T1-NS | -0.743 | 0.539 | 0.055 | -0.125 | -0.870 | -0.424 |
| T2-NS | 0.963 | 2.635 | 0.707 | 1.685 | -0.357 | -0.949 |
| T3-NS | 0.515 | 1.303 | -0.631 | -0.782 | -1.023 | 1.107 |


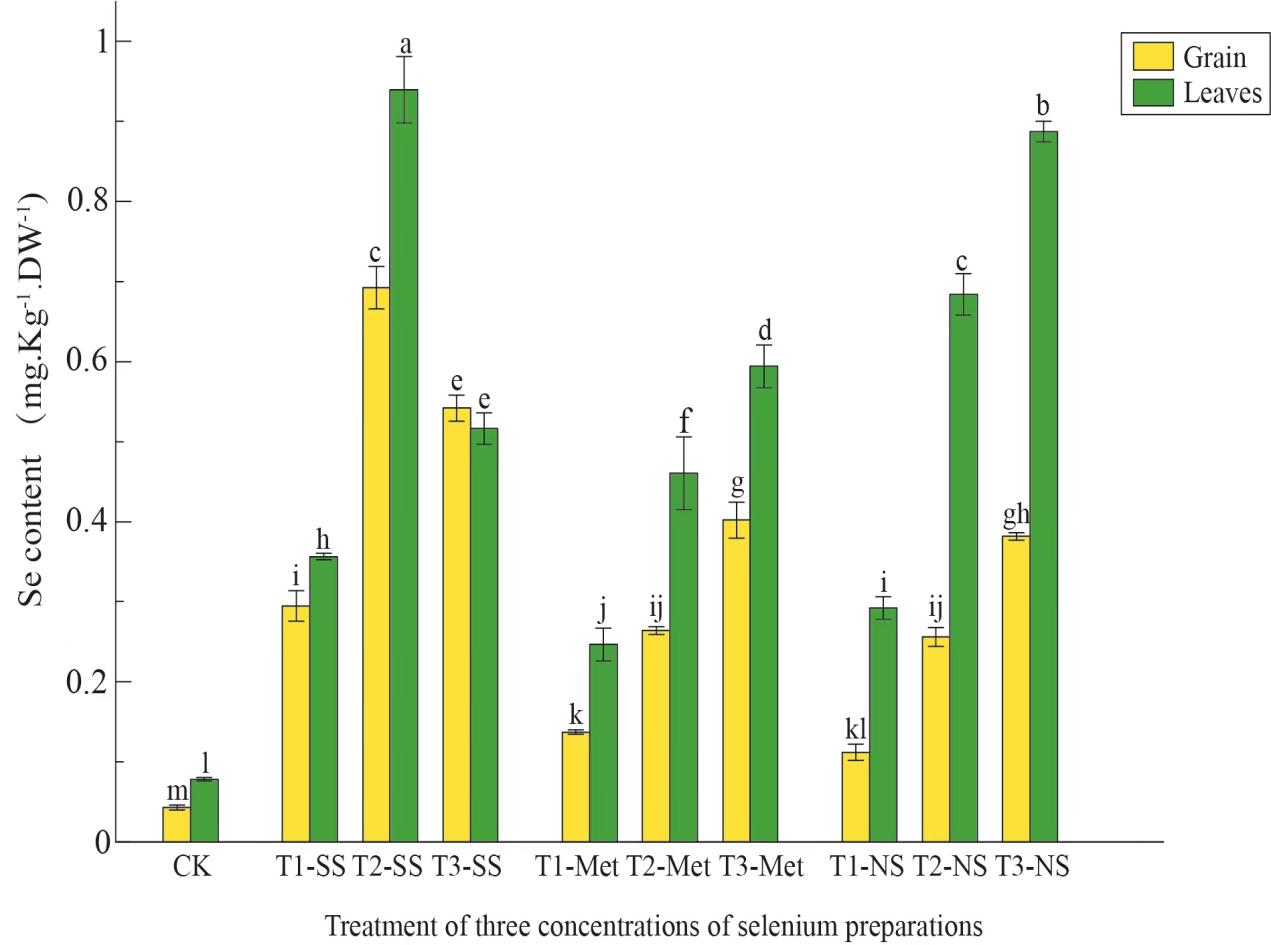


Fig S1 The Se content in leaves and grains spraying exogenous Se of LY in tillering stage
