# Supplementary material for: Enhancement of Nutrient, Trace Element, and Organic Selenium Contents of Ratooning Rice Grains and Straw Through Foliar Application of Selenite
Source: Foods. 2024 Nov 14;13(22):3637. doi: 10.3390/foods13223637 (PMC11594030; doi:10.3390/foods13223637)
Supplement: Supplementary file 1 [file foods-13-03637-s001.zip › 10-27-Table S2~S3.pdf]

Table S2. The correlation matrix eigenvalues and corresponding matrix eigenvectors of nutritional quality, trace elements and pigment content traits of RR.

Primary Component	Principal Component Number					
	1	2	3	4	5	6
Eigenvalue	2.279	1.417	1.188	1.033	0.752	0.675
Percentage of variance (%)	47.203%	18.252%	12.830%	9.696%	5.140%	4.137%
Cumulative (%)	47.203%	65.455%	78.285%	87.981%	93.121%	97.258%
Se	3.216	−0.254	0.193	−0.021	0.488	0.265
Organic Se (O-Se)	3.159	−0.176	0.198	−0.217	0.491	−0.780
Protein Se (P-Se)	3.227	−0.282	0.302	−0.305	0.334	−0.290
Zn	1.128	2.636	0.530	−0.575	−0.710	0.786
Cu	−0.841	−2.651	0.186	1.107	−0.844	−0.535
Mn	0.776	−1.184	2.800	−0.561	0.802	0.329
Fe	2.817	1.032	−0.605	1.166	−0.420	−0.134
Resistant starch (RS)	1.288	−1.260	−2.324	0.243	1.066	0.971
Amylose content (AC)	2.980	−0.070	−0.579	−0.621	−0.751	−0.892
Amino acid (AA)	−1.164	1.923	0.339	1.873	1.221	−0.820
Antioxidant activity (AnC)	−1.994	0.422	−0.994	−2.100	0.633	−0.896

Table S3. The principal component scores of nutritional quality, trace elements content of RR grains.

Eigenvalue	PC1	PC2	PC3	PC4	PC5	PC6
CK1	−2.661	−0.854	1.042	0.179	0.210	0.327
CK2	−2.691	−0.957	1.045	0.223	−0.190	−0.017
CK3	−2.773	−0.690	0.878	0.296	0.663	0.033
T1-SS	−0.196	1.330	0.370	−2.630	0.392	−0.372
T2-SS	4.442	−0.666	0.654	0.274	1.095	0.393
T3-SS	3.250	−0.666	0.947	−0.198	−0.005	−0.112
T1-MeSe	−1.435	1.166	−2.558	0.469	1.297	0.035
T2-MeSe	0.872	−2.591	−1.765	−0.270	−0.600	−1.079
T3-MeSe	0.456	−0.549	−0.744	0.878	−0.612	1.059
T1-NS	−0.743	0.539	0.055	−0.125	−0.870	−0.424
T2-NS	0.963	2.635	0.707	1.685	−0.357	−0.949
T3-NS	0.515	1.303	−0.631	−0.782	−1.023	1.107

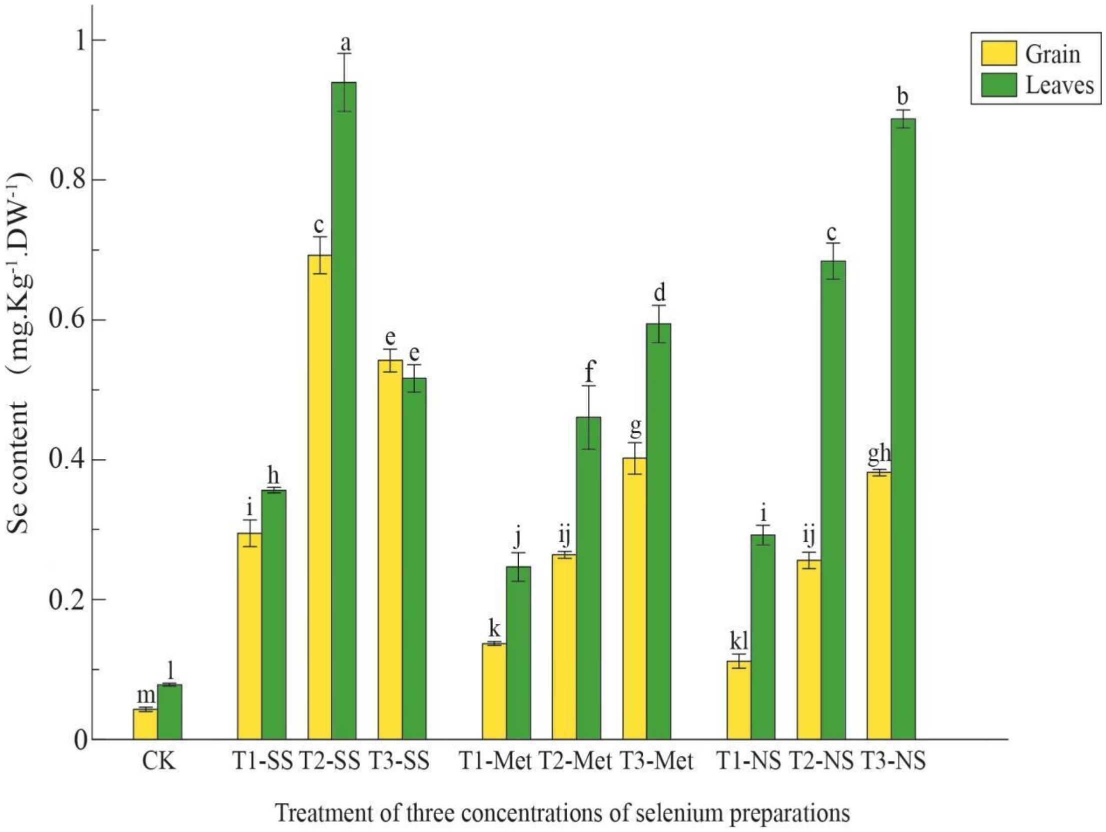

Figure S1. The Se content in leaves and grains spraying exogenous Se of LY in tillering stage.
