# Supplementary material for: Enhancement of Nutrient, Trace Element, and Organic Selenium Contents of Ratooning Rice Grains and Straw Through Foliar Application of Selenite
Source: Foods. 2024 Nov 14;13(22):3637. doi: 10.3390/foods13223637 (PMC11594030; doi:10.3390/foods13223637)
Supplement: Supplementary file 1 [file foods-13-03637-s001.zip › 10-27-Table S1.pdf]

Table S1. The content of Se speciation forms and total Se in rice leaves and grains ($mg \cdot (kg\ D.W.)^{-1}$).

Treatment	Total Se content		SeMet		SeCys2	
	leaves	grain	leaves	grain	leaves	grain
CK	0.078 ± 0.041 e	0.063 ± 0.016 f	0.0613 ± 0.012 g	0.046 ± 0.003 f	0.012 ± 0.007 d	0.098 ± 0.021 b
T1-SS	0.356 ± 0.053 c	0.259 ± 0.113 d	0.290 ± 0.018 e	0.251 ± 0.036 d	0.056 ± 0.029 b	0.036 ± 0.006 c
T2-SS	0.929 ± 0.125 a	0.757 ± 0.131 a	0.763 ± 0.044 a	0.660 ± 0.044 b	0.151 ± 0.061 a	0.089 ± 0.024 b
T3-SS	0.506 ± 0.112 b	0.342 ± 0.067 c	0.402 ± 0.035 d	0.411 ± 0.083 c	0.069 ± 0.041 a	0.099 ± 0.011 b
T1-SeMet	0.247 ± 0.033 d	0.137 ± 0.016 e	0.194 ± 0.037 f	0.121 ± 0.015 e	0.031 ± 0.012 c	0.014 ± 0.001 e
T2-SeMet	0.461 ± 0.053 b	0.402 ± 0.081 b	0.397 ± 0.066 d	0.372 ± 0.085 c	0.029 ± 0.006 c	0.011 ± 0.001 e
T3-SeMet	0.594 ± 0.094 b	0.262 ± 0.068 d	0.495 ± 0.065 c	0.229 ± 0.071 d	0.082 ± 0.011 b	0.024 ± 0.011 d
T1-NS	0.288 ± 0.031 d	0.112 ± 0.011 e	0.206 ± 0.053 f	0.804 ± 0.061 a	0.042 ± 0.012 bc	0.009 ± 0.000 e
T2-NS	0.684 ± 0.101 b	0.382 ± 0.023 c	0.503 ± 0.056 c	0.285 ± 0.032 d	0.105 ± 0.031 b	0.578 ± 0.045 a
T3-NS	0.887 ± 0.153 a	0.256 ± 0.042 d	0.624 ± 0.121 b	0.177 ± 0.022 e	0.176 ± 0.013 a	0.471 ± 0.031 a

Note: Means followed by different letters indicate significant differences ($p < 0.05$). Control—without application of Se.

(Continued table)

Treatment	SeMeCys		Se (IV)		Se(VI)	
	leaves	grain	leaves	grain	leaves	grain
CK	0.000 ± 0.000 b	0.000 ± 0.000 c	0.047 ± 0.011 a	0.070 ± 0.002 e	0.000 ± 0.000 d	0.000 ± 0.000 b
T1-SS	0.000 ± 0.000 b	0.000 ± 0.000 c	0.001 ± 0.000 d	0.085 ± 0.004 d	0.000 ± 0.000	0.000 ± 0.000 b
T2-SS	0.000 ± 0.000 b	0.000 ± 0.000 c	0.015 ± 0.003 c	0.079 ± 0.014 d	0.000 ± 0.000 d	0.000 ± 0.000 b
T3-SS	0.000 ± 0.000 b	0.000 ± 0.000 c	0.045 ± 0.005 a	0.324 ± 0.031 a	0.000 ± 0.000 d	0.000 ± 0.000 b
T1-SeMet	0.000 ± 0.000 b	0.000 ± 0.000 c	0.022 ± 0.007 b	0.019 ± 0.011 g	0.000 ± 0.000 d	0.000 ± 0.000 b
T2-SeMet	0.016 ± 0.001 a	0.009 ± 0.000 a	0.019 ± 0.003 b	0.102 ± 0.014 c	0.000 ± 0.000 d	0.000 ± 0.000 b
T3-SeMet	0.014 ± 0.001 a	0.004 ± 0.000 b	0.028 ± 0.011 b	0.054 ± 0.008 f	0.000 ± 0.000 d	0.000 ± 0.000 b
T1-NS	0.000 ± 0.000 b	0.000 ± 0.000 c	0.016 ± 0.007 c	0.052 ± 0.004 f	0.023 ± 0.001 c	0.014 ± 0.005 a
T2-NS	0.000 ± 0.000 b	0.000 ± 0.000 c	0.044 ± 0.012 a	0.291 ± 0.033 a	0.033 ± 0.004 b	0.011 ± 0.003 a
T3-NS	0.000 ± 0.000 b	0.000 ± 0.000 c	0.043 ± 0.011 a	0.201 ± 0.016 b	0.043 ± 0.008 a	0.012 ± 0.002 a
